# New tools for evaluating protein tyrosine sulfation: tyrosylprotein sulfotransferases (TPSTs) are novel targets for RAF protein kinase inhibitors

**DOI:** 10.1042/BCJ20180266

**Published:** 2018-08-14

**Authors:** Dominic P. Byrne, Yong Li, Pawin Ngamlert, Krithika Ramakrishnan, Claire E. Eyers, Carrow Wells, David H. Drewry, William J. Zuercher, Neil G. Berry, David G. Fernig, Patrick A. Eyers

**Affiliations:** 1 Department of Biochemistry, Institute of Integrative Biology, University of Liverpool, Liverpool L69 7ZB, U.K.; 2 Centre for Proteome Research, Institute of Integrative Biology, University of Liverpool, Liverpool L69 7ZB, U.K.; 3 Structural Genomics Consortium, UNC Eshelman School of Pharmacy, University of North Carolina at Chapel Hill, Chapel Hill, NC 27599, U.S.A.; 4 Lineberger Comprehensive Cancer Center, University of North Carolina at Chapel Hill, Chapel Hill, NC 27599, U.S.A.; 5 Department of Chemistry, University of Liverpool, Liverpool L69 7ZD, U.K.

**Keywords:** kinase inhibitor, PAPS, RAF, sulfation, sulfotransferase, tyrosine sulfotransferase

## Abstract

Protein tyrosine sulfation is a post-translational modification best known for regulating extracellular protein–protein interactions. Tyrosine sulfation is catalysed by two Golgi-resident enzymes termed tyrosylprotein sulfotransferases (TPSTs) 1 and 2, which transfer sulfate from the cofactor PAPS (3′-phosphoadenosine 5′-phosphosulfate) to a context-dependent tyrosine in a protein substrate. A lack of quantitative tyrosine sulfation assays has hampered the development of chemical biology approaches for the identification of small-molecule inhibitors of tyrosine sulfation. In the present paper, we describe the development of a non-radioactive mobility-based enzymatic assay for TPST1 and TPST2, through which the tyrosine sulfation of synthetic fluorescent peptides can be rapidly quantified. We exploit ligand binding and inhibitor screens to uncover a susceptibility of TPST1 and TPST2 to different classes of small molecules, including the anti-angiogenic compound suramin and the kinase inhibitor rottlerin. By screening the Published Kinase Inhibitor Set, we identified oxindole-based inhibitors of the Ser/Thr kinase RAF (rapidly accelerated fibrosarcoma) as low-micromolar inhibitors of TPST1 and TPST2. Interestingly, unrelated RAF inhibitors, exemplified by the dual BRAF/VEGFR2 inhibitor RAF265, were also TPST inhibitors *in vitro*. We propose that target-validated protein kinase inhibitors could be repurposed, or redesigned, as more-specific TPST inhibitors to help evaluate the sulfotyrosyl proteome. Finally, we speculate that mechanistic inhibition of cellular tyrosine sulfation might be relevant to some of the phenotypes observed in cells exposed to anionic TPST ligands and RAF protein kinase inhibitors.

## Introduction

Like tyrosine phosphorylation [1], reversible tyrosine sulfation is a critical covalent modification that occurs post-translationally on proteins [2]. Originally identified more than half a century ago in sulfated fibrinogen and gastrin [3], tyrosine sulfation occurs on a wide range of secreted polypeptides in multicellular eukaryotes and involves the transfer of a negatively charged sulfate group from the sulfate donor PAPS (3′-phosphoadenosine-5′-phosphosulfate) to a phenolic tyrosine residue. Tyrosine sulfation is catalysed by two Golgi-associated membrane enzymes termed tyrosylprotein sulfotransferases 1 and 2 (TPST1 and 2), and sulfation leads to biologically relevant changes in a large number of protein activities [2]. For example, sulfation can change the affinity of extracellular protein–protein interactions, such as those involved in chemotaxis [4], and host–pathogen interactions [5]. It also controls the proteolytic processing of both bioactive peptides [6,7] and secreted antibodies [8], and multi-site tyrosine sulfation can change the function of several blood-coagulation regulators, including factor VIII [9,10]. Interest in the pathophysiological analysis and therapeutic targeting of tyrosine sulfation was heightened by the finding that N-terminal chemokine receptor tyrosine sulfation in the HIV G-protein coupled receptor CCR5 [11,12] plays a crucial role in coat binding and viral infection. Earlier studies had implicated tyrosine sulfation in the proteolytic control of the complement cascade component through decreased activity of C4 [13], the generation of gastrin from progastrin [14], and in regulating the binding of amino-terminal sulfated P-selectin glycoprotein ligand-1 (PSGL-1) to P-selectin [15]. Interestingly, the binding of l-selectin on lymphocytes to mucin-like glycoproteins on endothelial cells is also regulated by sulfation, although the sialyl Lewis^X^ surface antigen is modified by a distinct carbohydrate 6-*O* sulfotransferase [16].

TPST1 was originally purified from bovine adrenal medulla [17,18], and distinct human genes encoding TPST1 and TPST2 have been cloned [19], with expression patterns varying markedly in both cells and tissues [20–22]. Both enzymes are believed to reside in the trans-Golgi compartment of the secretory pathway, and as type II transmembrane-containing enzymes with >85% sequence similarity in the intracellular catalytic domains, which are luminal-facing for substrate modification [19,21,22]. TPSTs interact with the sulfate-donor cofactor PAPS and an appropriate (often acidic) tyrosine-containing protein substrate. Recent experiments suggest that TPST1 and TPST2 might function as homo- or heterodimers [23,24], providing regulatory opportunities for the control of site-specific sulfation among substrates. In general, tyrosine sulfation occurs in an acidic context in proteins and model substrates [2,18,24–26], although some, including the bioactive protein gastrin, lack acid residues adjacent to the site of sulfation [14]. Analysis of a variety of synthetic peptides and intact proteins confirms that TPST1 and TPST2 can also control site-specific sulfation on multiple tyrosine residues, which are often clustered, consistent with a processive mechanism of modification [7,27], or directionally distributed towards the substrate N-terminus [20,28]. Crystal structures of TPST1 complexed with substrate peptides that are sulfated with different efficiencies have also been reported, and comparative analysis suggests differential substrate preferences for acidic residues adjacent to the site of modification [24,29]. Structural comparison suggests a shared catalytic mechanism and substrate-binding energetics, driven by charge-based dynamic interactions. Bioinformatic analysis hints at a substantial and complex tyrosine sulfoproteome [30,31], hence uncovering the extent, substrate determinants and biological function of tyrosine-sulfated proteins remains a high-priority technical challenge for mass spectrometry (MS)-based proteomics [32].

The analysis of tyrosine sulfation currently relies heavily on genetic and relatively low-throughput MS-based analysis, and only a few low-affinity inhibitors of TPSTs have been reported [33–35]. Moreover, due to a lack of chemical tool compounds, biological sulfation remains an understudied process relying on non-specific cytotoxic compounds, such as chlorate, to induce non-specific effects on sulfation [36]. The similarity between the sulfotransferase cofactor PAPS and the phosphate donor ATP (utilised by protein kinases) raises questions as to whether PAPS-dependent sulfotransferases might be broad inhibitory targets for new or repurposed small molecules that target nucleotide-binding sites, especially well-studied families of compounds such as protein kinase inhibitors. Moreover, the mode of substrate peptide recognition observed in substrate- and cofactor-bound TPST2 structures closely resembles that established for the insulin-receptor tyrosine kinase bound to a tyrosine-containing (YMXM) substrate and an ATP analogue [37], inviting further comparison between TPSTs and the highly druggable protein kinase superfamily [38,39].

Analysis of TPST-based catalysis using small molecules remains in its infancy and is currently hampered by a lack of rapid, flexible, and reliable assays with which to screen for suitable inhibitors. Conventional procedures employ ^35^S-based detection of sulfated tyrosine in synthetic peptides [18,21,34] or, increasingly, rely on gas-phase MS-based detection of sulfated peptides [35,40,41]. Both of these approaches have technical drawbacks, and can be time-consuming, although ^35^S-based peptide sulfation by TPST2 was used to discover the first low-affinity reversible TPST2 inhibitors from a combinatorial library of aldehyde-linked heterocyclic compounds [34]. Recently, indirect fluorescent assays have been reported, including a PAPS depletion/reconstitution approach to monitor sulfate transfer [28] and continuous TPST1 and 2 assays reporting fluorescence-induced peptide sulfation [35]. The latter approach monitors peptide sulfation over relatively long time periods and requires inflexible positioning of the fluorophore relative to the modified tyrosine and flanking amino acid sulfation determinants. Nonetheless, such assays can be employed to discover small-molecule inhibitors in screens, with several anionic compounds recently identified and cross-validated from commercial libraries [33,35].

In the present paper, we describe differential scanning fluorimetry (DSF) and sulfation assays that permit real-time analysis of TPST1- and TPST2-mediated peptide sulfation, allowing us to evaluate TPST interactions with a variety of ligands and small-molecule inhibitors. PAPS-dependent sulfation of peptides leads to a charge-induced mobility change, driven through intrinsic properties of a sulfotyrosine-containing substrate. Sulfation is detected by a real-time mobility shift using a fluorescent microfluidic assay originally developed for the detection of peptide tyrosine phosphorylation [42]. In conjunction with analytical DSF, we identified a variety of known ligands as new TPST1 and TPST2 inhibitors, including the promiscuous protein kinase inhibitor rottlerin and a family of oxindole-based RAF (rapidly accelerated fibrosarcoma) kinase inhibitors from the Published Kinase Inhibitor Set (PKIS). In a related paper, published back-to-back with the present study, we demonstrate that some of these compounds also inhibit the oligosaccharide sulfotransferase activity of heparan sulfate 2-*O* sulfotransferase [43], a related PAPS-dependent enzyme. Finally, chemically distinct inhibitors with activity towards the proto-oncogenic kinase RAF, exemplified by the dual BRAF/VEGFR2 inhibitor RAF265 (CHIR-265), were discovered to be more specific TPST inhibitors *in vitro*. We propose that kinase inhibitors might be discovered through further screening, by chemical repurposing, or even redesigned to create new classes of TPST inhibitor. Moreover, we speculate that inhibition of cellular tyrosine sulfation by some of the compounds evaluated in the present study might contribute to the phenotypes observed in cells exposed to RAF kinase inhibitors.

## Experimental

### Materials and methods

#### Chemicals and compounds

All standard biochemicals were purchased from either Melford or Sigma and were of the highest analytical quality that could be obtained. PAPS (adenosine 3′-phosphate 5′-phosphosulfate, lithium salt hydrate), APS (adenosine 5′-phosphosulfate, sodium salt), PAP (adenosine 3′–5′-diphosphate, disodium salt), CoA (coenzyme A, sodium salt) dephosphoCoA (3′-dephosphoCoA, sodium salt hydrate), ATP (adenosine 5′-triphosphate, disodium salt hydrate), ADP (adenosine 5′-diphosphate, disodium salt), AMP (adenosine 5′-monophosphate, sodium salt), GTP (guanosine 5′-triphosphate, sodium salt hydrate), or cAMP (adenosine 3′,5′-cyclic monophosphate, sodium salt) were all purchased from Sigma and stored at −80°C to ensure maximal stability. Rottlerin, suramin, aurintricarboxylic acid, and all named kinase inhibitors were purchased from either Sigma, BD laboratories, Selleck, or Tocris.

#### Cloning, protein purification, and protein analysis

DNA encoding human TPST1 (residues Lys43–Leu360) and TPST2 (residues Gly43–Leu359) enzymes lacking the transmembrane domains was amplified by PCR and cloned into pOPINF (OPPF-UK) to produce recombinant protein containing an N-terminal 6xHis tag and a 3C protease cleavage site. Recombinant TPST1 and TPST2 proteins were expressed in BL21 (DE3) pLysS *Escherichia coli* (Novagen); expression was induced with 0.4 mM IPTG for 18 h at 37°C, and protein was isolated from inclusion bodies and refolded as described [44]. In brief, cells were resuspended in 3 ml of ice-cold lysis buffer [50 mM Tris–Cl (pH 8.0), 10 mM MgCl_2_, 1 mM DTT supplemented with cOmplete, EDTA-free, protease inhibitor cocktail tablets (Roche)] per gram of *E. coli* cell pellet and flash-frozen with liquid nitrogen. Cells were disrupted by sonication; inclusion bodies were collected by centrifugation for 1 h at 10 000x***g*** at 4°C and washed in ice-cold WB1 (50 mM Tris–Cl (pH 8.0), 100 mM NaCl, 10 mM EDTA, and 1% (v/v) Triton X-100) followed by WB2 (20 mM Tris–Cl (pH 8.0), 200 mM NaCl, and 1 mM EDTA). Inclusion bodies were resuspended in SB (100 mM Tris–Cl (pH 8.0), 6 M guanidine hydrochloride, 5 mM EDTA, and 10 mM DTT) and incubated at 4°C with constant agitation. SB buffer was supplemented with fresh DTT (10 mM DTT) after 12 h and incubated for 2 h at room temperature. Insoluble material was removed by centrifugation (1 h, 60 000x***g***, 4°C), and the supernatant was concentrated by ultrafiltration (Amicon Ultra-15 centrifugal filter unit, 10 kDa cut-off) and then diluted 10-fold with buffer A (100 mM Na-acetate (pH 4.5), 6 M guanidine hydrochloride, and 10 mM DTT). A 5 ml aliquot of concentrated TPST (∼150 mg) was slowly added (using a peristaltic pump) while mixing with a magnetic stirrer to 1 l of pre-chilled refolding buffer, which comprised 50 mM Tris–Cl (pH 8.5), 500 mM guanidine hydrochloride, 10 mM NaCl, 0.4 mM KCl, 0.1 mM EDTA, 0.14 mM *n*-dodecyl β-D-maltoside, 5 mM reduced glutathione (GSH), and 2.5 mM oxidised glutathione (GSSG). The refolding mixture was then incubated for 20 h at 4°C without mixing and precipitated protein was subsequently removed by centrifugation. Soluble TPST protein was then purified by sequential immobilised metal affinity chromatography and size-exclusion chromatography (SEC) using a HiLoad 16/600 Superdex200 column (GE Healthcare) equilibrated in 50 mM Tris–Cl (pH 7.4), 100 mM NaCl, and 10% (v/v) glycerol. Glutathione-*S*-transferase (GST)-tagged CC4-tide (EDFED**Y**EFDG**)** was cloned into pOPINJ (OPPF-UK) and affinity-purified from BL21 (DE3) pLysS *E. coli* using Glutathione Sepharose 4B (GE Healthcare) and SEC. The tyrosine kinase EphA3, comprising the kinase domain and the juxtamembrane region with an N-terminal 6xHis-tag, was expressed in pLysS *E. coli* from pET28a LIC, and purified using Ni-NTA agarose and gel filtration, as described recently [42]. Halo-FGF7 was purified as previously described [45].

#### SDS–PAGE and immunoblotting

After the assay, proteins were denatured in Laemmli sample buffer, heated at 95°C for 5 min and then analysed by SDS–PAGE with 10% (v/v) polyacrylamide gels. Gels were stained and destained using a standard Coomassie Brilliant Blue protocol. To evaluate protein sulfation and phosphorylation by immunoblotting, standard western blotting procedures were followed using monoclonal anti-sulfotyrosine antibody clone Sulfo-1C-A2 (Millipore) generated using a phage display procedure and sulfotyrosine selection peptide antigens [46] in the presence of appropriate positive and negative controls, and modifications visualised using the ECL reagent.

#### Differential scanning fluorimetry assays

Thermal shift/stability assays (TSAs) were performed with a StepOnePlus Real-Time PCR machine (Life Technologies) using the Sypro-Orange dye (Invitrogen) and thermal ramping between 20 and 95°C in 0.3°C step intervals per data point to induce denaturation in the presence or absence of various biochemical and small-molecule inhibitors [47,48]. TPST1 and TPST2 were assayed at a final concentration of 5 µM in 50 mM Tris–HCl (pH 7.4) and 100 mM NaCl. The final DMSO concentration in the presence or absence of the indicated concentrations of ligand was no higher than 4% (v/v). None of the test compounds analysed in the absence of TPSTs were found to interfere with fluorescent detection of Sypro-Orange binding. Normalised data were processed using the Boltzmann equation to generate sigmoidal denaturation curves, and average *T*_m_/Δ*T*_m_ values were calculated as described [48,49] using the GraphPad Prism software.

#### EZ Reader II-based peptide sulfation assays

Fluorescently tagged peptides used in TPST sulfotransferase assays were derived from the human physiological substrate sequences where noted. A 5-FAM fluorophore, with a maximal absorbance of 495 nm and a maximal emission absorbance of 520 nm that could be detected in an EZ Reader via LED-induced fluorescence, was covalently coupled to the free N-terminus of each peptide. CC4-tide (5-FAM-EDFED**Y**EFDG-CONH_2_ and the equivalent peptide lacking the single acceptor tyrosine residue, 5-FAM-EDFED**F**EFDG-CONH_2_), were modified from the human Complement C4 protein [13], fibroblast growth factor 7 (FGF7, 5-FAM-ERHTRSYD**Y**MEGGD-CONH_2_), and C–C motif chemokine receptor 8 (CCR8, 5-FAM-TTVTD**Y**YYPDIFSS-CONH_2_), and P-selectin glycoprotein ligand-1 (PSGL1, 5-FAM-TEYEYLD**Y**DFLPETE-CONH_2_) peptides were derived from the appropriate human sequences (site of tyrosine sulfation shaded in bold). Peptides were synthesised using solid-phase Fmoc chemistry and after HPLC purification (>95%), the expected intact peptide mass was confirmed by MALDI-TOF mass spectrometry (Pepceuticals, Leicester, U.K.). The PerkinElmer LabChip EZ II Reader system [50], 12-sipper chip and CR8 coating, assay separation buffer, and a synthetic fluorescent Ephrin A3 substrate (Ephrin A3-tide, 5-FAM-EFPIYDLPAKK-CONH_2_) were all purchased from PerkinElmer. Pressure and voltage settings were adjusted manually to afford optimal separation of tyrosine-sulfated and non-sulfated peptides. Individual sulfation assays were performed in a 384-well plate in a volume of 80 μl in the presence of the indicated concentration of PAPS (Sigma–Aldrich), 50 mM HEPES (pH 7.4), 0.015% (v/v) Brij-35, and 5 mM MgCl_2_ (unless specified otherwise), and the degree of peptide sulfation was directly calculated by the EZ Reader software by differentiating sulfopeptide : peptide ratios. The activity of TPST proteins in the presence of inhibitors was quantified by monitoring the amount of sulfopeptide generated over the assay time relative to the control assay with no additional inhibitor molecule. Data were normalised with respect to these control assays, with sulfate incorporation into the peptide limited to ∼20% to prevent depletion of PAPS and to ensure assay linearity. *K*_m_ and IC_50_ values were determined by nonlinear regression analysis using the Graphpad Prism software

#### Biochemical and small-molecule screening by DSF and TPST enzyme assay

The PKIS chemical library (designated with SB, GSK, or GW prefixes) comprising 367 largely ATP-competitive kinase inhibitors, covering 31 chemotypes originally knowingly designed to inhibit 24 distinct protein kinases [51,52], was stored frozen as a 10 mM stock in DMSO at −80°C. This inhibitor library is characterised as highly drug-like (∼70% with molecular mass <500 Da and clogP values <5). For initial screening, compounds pre-dissolved in DMSO were pre-incubated with TPST1 or TPST2 for 10 min, and sulfotransferase reactions were initiated by the addition of the universal sulfate donor PAPS. For inhibition assays, competition assays, or individual IC_50_ value determination, the appropriate compound range was prepared by serial dilution in the appropriate solvent and added directly into the assay to the indicated final concentration. All control experiments contained 4% (v/v) DMSO.

#### Molecular docking analysis

Rottlerin, GW305074X, suramin, and RAF265 were built using Spartan16 (https://www.wavefun.com) and energy-minimised using the Merck molecular forcefield. GOLD 5.2 (CCDC Software;) was used to dock molecules [53], with the binding site defined as 10 Å around the 5′-phosphorous atom of PAP, using co-ordinates from human TPST1 PDB ID: 5WRI [24]. A generic algorithm with ChemPLP as the fitness function [54] was used to generate 10 binding-modes per ligand in HS2ST. Protons were added to the protein. Default settings were retained for the ‘ligand flexibility’ and ‘fitness and search options’; however, GA settings were changed manually to 200%.

## Results

### Analysis of human TPST1 and TPST2 using a reliable TSA

**Figure 1.**
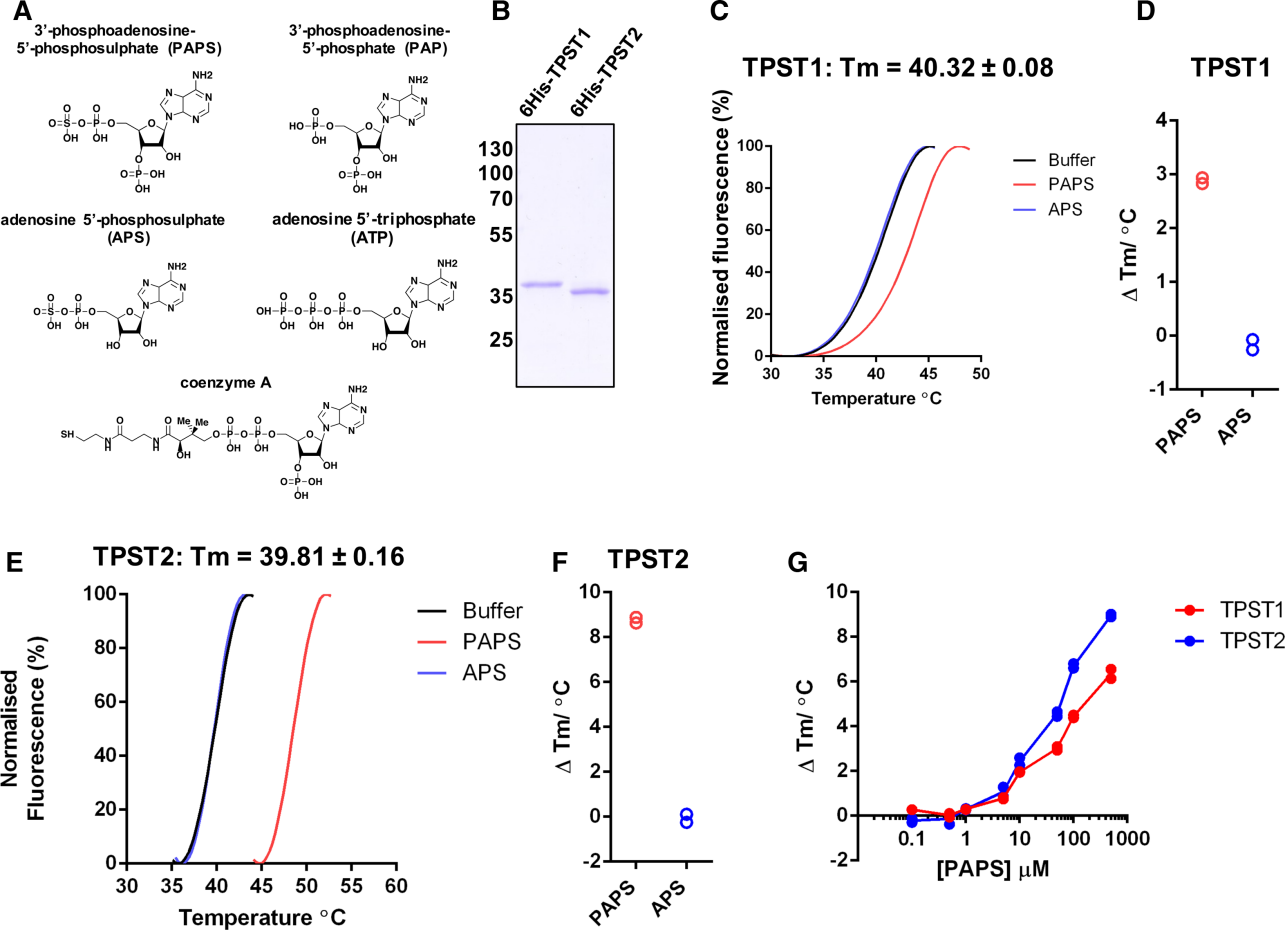
Analysis of purified recombinant 6His-TPST proteins. (**A**) Biochemical structure of PAPS- and PAPS-related compounds. (**B**) Coomassie blue staining of purified recombinant 6His-TPST enzymes: 1 µg of TPST1 and 2 were analysed by SDS–PAGE after purification to near homogeneity. (**C**) TSA, and calculation of *T*_m_, for TPST1 (5 µM) in the presence of 0.5 mM PAPS (red) or 0.5 mM APS (blue); buffer control is in black. (**D**) Δ*T*_m_ for TPST1 in the presence of PAPS and APS, as measured by DSF, data derived from (**C**). Δ*T*_m_ values were calculated by subtracting the control *T*_m_ value (buffer, no nucleotide) from the measured *T*_m_ value. (**E**) As for (**C**) but using TPST2. (**F**) As for (**D**), but employing TPST2. (**G**) Analysis of PAPS-dependent thermal stabilisation of TPST1 and TPST2. TSA employing TPST1 or TPST2 proteins (5 µM) was measured in the presence of the indicated concentration of PAPS. Δ*T*_m_ values were calculated by DSF, as described above.

To drive the development of new approaches to assay and inhibit protein tyrosine sulfation, we developed a DSF assay to examine the thermal stability of TPST1 or TPST2 in the presence or absence of biochemical ligands (Figure 1A). We purified recombinant soluble human 6His-tagged TPST1 and TPST2 catalytic domains (amino acids 43–360 and 43–559, respectively, lacking the transmembrane domain) from bacterial inclusion bodies to near homogeneity (Figure 1B). After refolding from guanidine hydrochloride into a Tris-based buffer, TPST thermal stability and unfolding profiles were measured in the presence of the known sulfated cofactor PAPS, or the dephosphorylated precursor APS, whose phosphorylation at the 3′-position on the adenine ring by APS kinase generates PAPS in cells. Heating of TPST1 and TPST2 generated a typical heat-induced unfolding profile with both TPST1 and TPST2 exhibiting almost identical *T*_m_ values (formally, the temperature at which 50% of the protein is unfolded based on fluorescence) of ∼40°C (Figure 1C,E). In both cases, inclusion of PAPS in the unfolding assay induced a shift in the *T*_m_ value, suggesting that both enzymes were folded and could bind to a physiological cofactor. In the case of TPST1, PAPS (but not APS) induced a Δ*T*_m_ value of ∼3°C (Figure 1C,D), whereas TPST2 stability shifted by ∼9°C in the presence of PAPS, but not APS (Figure 1E,F). A side-by-side comparison of TPST1 and TPST2 over a range of PAPS concentrations demonstrated concentration-dependent effects on TPST stability, with a more marked shift in TPST2 stability at all concentrations tested (Figure 1G). We next compared thermal unfolding in the presence of a panel of nucleotide-based cofactors. These experiments demonstrated a lack of significant thermal shift by Mg^2+^ ions, APS, AMP, or cAMP. In contrast, ADP, PAP, CoA, acetyl CoA, and GTP all induced marked stabilisation of both TPST1 and TPST2 at near stoichiometric concentrations in the assay, suggestive of high-affinity binding. In contrast with CoA, dephospho-CoA, which lacks a 3′-phosphoadenine group, was unable to induce thermal shifts in either TPST1 or 2, as established for APS, in which the 3′-phosphoadenine group is also absent. In the case of ATP, ADP, and GTP, TPST1 and TPST2 shifts were abolished in the presence of Mg^2+^ ions, presumably reflecting the very high affinity of this divalent cation for these nucleotides [48] (Supplementary Figure S1A,B).

### A novel microfluidic assay to quantify real-time peptide sulfation by TPST1 and TPST2

Thermal and enzymatic screening assays can generate complementary information to help evaluate ligand binding [55]. To extend our thermal analysis of TPST ligand binding to include real-time analysis of sulfate transfer, and help progress our eventual goal of discovering TPST1 and TPST2 inhibitors, we developed a novel enzyme assay for kinetic analysis of peptide tyrosine sulfation. The basic requirement of this assay was that it should report the enzymatic incorporation of sulfate onto a tyrosine residue of a synthetic peptide substrate with a high signal-to-noise ratio and be rapid, repeatable, and with relatively high throughput. Current protocols to monitor tyrosine sulfation generally involve ^35^S-based enzyme regeneration or intrinsic fluorescence assays, which are often unsuitable for kinetic or high-throughput analysis using different peptide substrates, and are prone to artefacts if compounds or cofactors that interfere with fluorescence detection are employed. However, as established below, our novel assay permits rapid real-time detection of non-radioactive sulfate incorporation into synthetic peptides.

### Synthetic peptides derived from human substrates are *in vitro* TPST1 and/or TPST2 substrates

**Figure 2.**
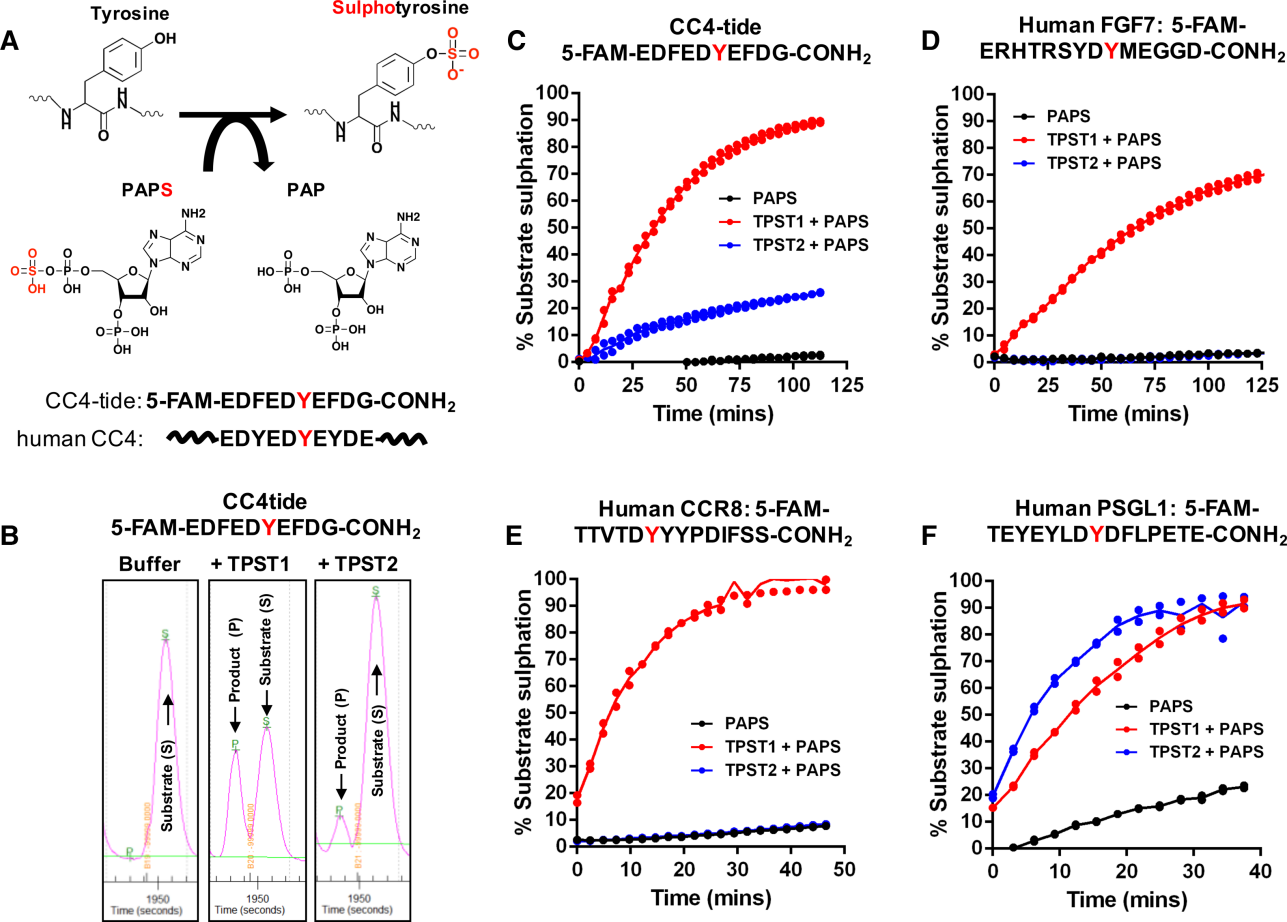
Detection of tyrosylprotein sulfotransferase activity using a direct microfluidic mobility shift assay and fluorescent peptide substrates. (**A**) Schematic representation of PAPS-dependent sulfate incorporation into a tyrosine residue of a substrate. The sequence of the synthetic single Tyr-containing peptide (CC4-tide, containing a fluorescent 5-FAM group at the N-terminus) is shown above the native human CC4 protein sequence. (**B**) TPST1 and TPST2-dependent tyrosine sulfation alters the microfluidic mobility of CC4-tide. Separation of the higher-mobility, sulfated (product, P) peptide from the lower-mobility (substrate, S) peptide occurs through a difference in their net charge. (**C–F**) Time-dependent tyrosine sulfation of CC4-tide (**C**) or fluorescently labelled tyrosine-containing substrate peptides derived from human FGF7 (**D**), CCR8 (**E**), or PSGL1 (**F**) proteins. Direct peptide sulfation was calculated by measuring the ratio of substrate peptide to sulfo-peptide at the indicated time points after adding the PAPS cofactor. All assays were performed at room temperature (20°C) using 2 µM final concentration of the appropriate fluorescent peptide substrate, 500 µM PAPS, and 0.4 µM TPST1 or TPST2 in the presence of 5 mM MgCl_2_.

To evaluate context-specific sulfation kinetics for TPST1 and TPST2, we synthesised a panel of peptides possessing tyrosine-containing sequences found in human proteins previously reported to be sulfated on tyrosine [2] and developed an assay to quantify peptide sulfation. The assay comprises a putative substrate peptide (containing a tyrosine in an acid context and culminating in an amide group), TPST1 or TPST2, and the PAPS cofactor (Figure 2A). To facilitate the detection of both sulfated and non-sulfated substrates in the same assay using a microfluidic platform, we appended an N-terminal fluorophore (5-FAM) to the peptide. Since tyrosylsulfate (singly charged under the assay conditions) and tyrosylphosphate (doubly charged) are chemically similar, and can potentially induce charge-based differences in peptide mobility when covalently attached to tyrosine, we reasoned that this assay would be able to detect sulfation in a similar way to that previously established for phosphorylation by Ser/Thr and Tyr kinases [42,56,57]. As shown in Figure 2B, incubation of a 5-FAM-conjugated 10-mer tyrosine-containing peptide from human complement C4 protein with TPSTs led to the appearance of an electrophoretically distinct product (P) when compared with the unmodified substrate (S). Different ratios of product to substrate were detected when TPST1 or TPST2 was included in the assay, but no product was detected with buffer and PAPS alone, suggesting that the new product was a tyrosine-sulfated peptide (Figure 2B).

We next compared the ability of TPST1 and TPST2 to modify the CC4 peptide (termed hereafter CC4-tide) in a kinetic assay, monitoring the real-time appearance of the sulfated peptide by detecting the increase in product peak height in a duplicate assay format. As shown in Figure 2C, TPST1 was much more efficient at modifying CC4-tide, inducing near-stoichiometric modification after 1 h. The rate of CC4-tide sulfation by TPST1 was responsive to divalent cations and could be increased 6- to 10-fold by including Mg^2+^ ions or Mn^2+^ ions in the buffer (Supplementary Figure S3A), despite a lack of detectable Mg^2+^ binding to TPST1 (or TPST2) by DSF (Supplementary Figure S1A,B), consistent with previous studies [18,28,35]. In contrast, over the same time and at the same concentration in the assay, purified TPST2 only sulfated CC4-tide to a stoichiometry of ∼20%. We next assessed whether TPST1 or TPST2 sulfated a fluorescent 14-mer peptide derived from human FGF7, which contains a single known site of tyrosine sulfation corresponding to Tyr27 in the mature growth factor [58]. Using this new assay, we were unable to detect FGF7-tide sulfation by TPST2, although TPST1 induced ∼70% peptide sulfation over the assay time course (Figure 2D). Interestingly, CCR8-tide, which was derived from the human CCR8 sequence, was even more rapidly sulfated by TPST1 than FGF7-tide, although it was not modified noticeably by TPST2 (Figure 2E). In marked contrast, a distinct fluorescent 14-mer sequence derived from human PSGL1 was rapidly, and stoichiometrically sulfated by both TPST1 and TPST2 (Figure 2F), confirming that these enzymes possess overlapping, as well as distinct, substrate specificities *in vitro*. We also found that the rate of TPST1-catalysed sulfation of FGF7, CCR8 and, to a much smaller extent, PSGL1 was enhanced by divalent cations, with a clear activating preference for Mn^2+^ ions (Supplementary Figure S3B–D). In contrast, and as established for CC4-tide, TSPT2 was essentially inactive towards FGF7 and CCR8 in the presence and absence of divalent cations, although divalent Mn^2+^ ions and, to a lesser extent, Mg^2+^ ions activated TPST2 (and TPST1) when PSGL1 was employed as the substrate (Supplementary Figure S3B–D).

As a test of the suitability of our assay to derive a reported kinetic parameter, we employed an appropriate concentration of TPST1 and TPST2, so that the degree of peptide sulfation was linear over the time course of the reaction. Under these conditions, the *K*_m_ value for PAPS in a TPST1-dependent CC4-tide assay was 6.6 ± 1.9 μM (Supplementary Figure S2), consistent with previous literature reports of 2–5 μM for TPST1 [18,25] or 12 μM for TPST2 obtained from transfected CHO cell medium [34] or ∼5 μM for recombinant TPST2 isolated from baculovirus-infected insect cells [35].

### Substrate and cofactor specificity for model TPST1 and TPST2 substrates

**Figure 3.**
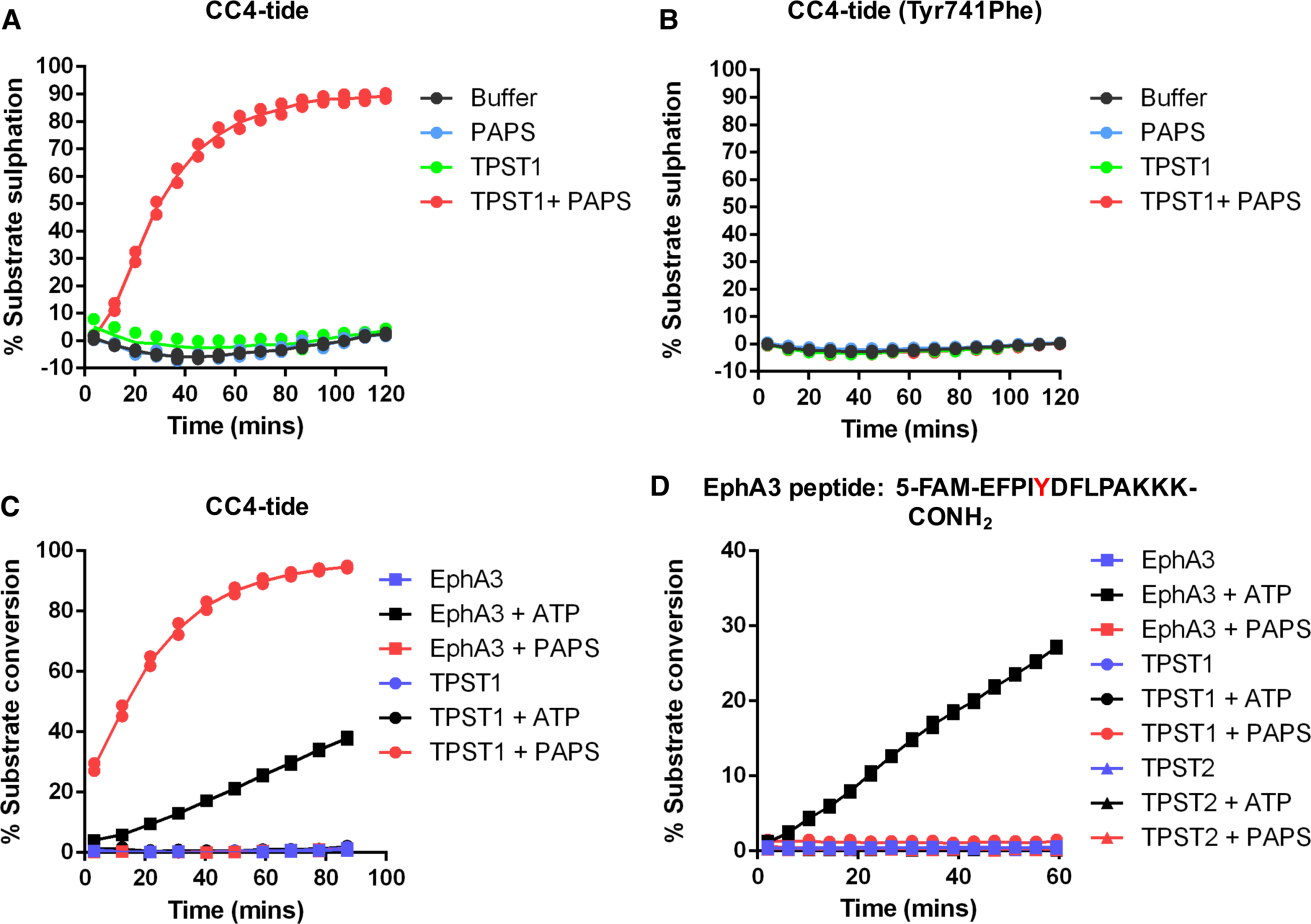
Changes in fluorescent peptide mobility are a consequence of TPST-catalysed tyrosine sulfation. Mobility analysis of TPST1-dependent peptide sulfation for (**A**) CC4-tide or (**B**) CC4-tide in which the Tyr acceptor site is mutated to Phe (Tyr741Phe). Recombinant TPST1 enzyme (0.4 µM) was assayed using 2 µM peptide substrate ± 10 µM PAPS as sulfate donor. (**C**) Dual detection of tyrosine phosphorylation or tyrosine sulfation of CC4-tide. TPST1 (0.2 µM) or EphA3 tyrosine kinase (0.3 µM) were incubated with 2 µM CC4-tide in the presence of 500 µM PAPS (sulfate donor) or 500 µM ATP (phosphate donor). (**D**) Lack of tyrosine sulfation of a distinct tyrosine-containing EphA3 substrate peptide by TPST1 or 2. Assay conditions were as for (**C**).

To investigate TPST site specificity, we confirmed that the single Tyr residue in CC4 represents the site of covalent modification identified in the mobility assay, by generating a peptide in which Tyr was substituted for a chemically analogous, but non-sulfatable, Phe residue. As shown in Figure 3A,B, PAPS-dependent CC4-tide sulfation probably occurs on Tyr, because the Phe-substituted peptide was not modified by incubation with TPST1 (or TPST2). To confirm sulfate and phosphate cofactor specificity in the assay, we evaluated sulfation and phosphorylation of the same CC4-tide substrate using either TPST1 or the tyrosine kinase Ephrin A3. Importantly, Ephrin A3 generated a modified (phosphorylated) CC4 product peptide in the presence of ATP, but not PAPS, whereas TPST1 generated a modified (sulfated) product peptide only in the presence of PAPS, but not ATP (Figure 3C). The electrophoretic mobility of sulfated (TPST1-generated) or phosphorylated (EphA3-generated) CC4 peptides relative to the non-modified peptide was very similar in our microfluidic assay conditions, consistent with similar physiochemical properties of anionic sulfotyrosine and phosphotyrosine formed in the assay. Finally, we confirmed the reported preference for tyrosine sulfation of peptide substrates in the context of an N-terminal acidic residue (which is targeted electrostatically to the cationic active site) by showing that a tyrosine kinase (TK) peptide substrate optimised for EphA3 phosphorylation was not modified by TPST1 or TPST2, presumably because it lacked an acidic residue at the –1 position relative to Tyr, although this did not prevent phosphorylation by EphA3 kinase in the presence of Mg-ATP (Figure 3D).

### TPST1 and TPST2 sulfate tyrosine in recombinant sulfoacceptor proteins

**Figure 4.**
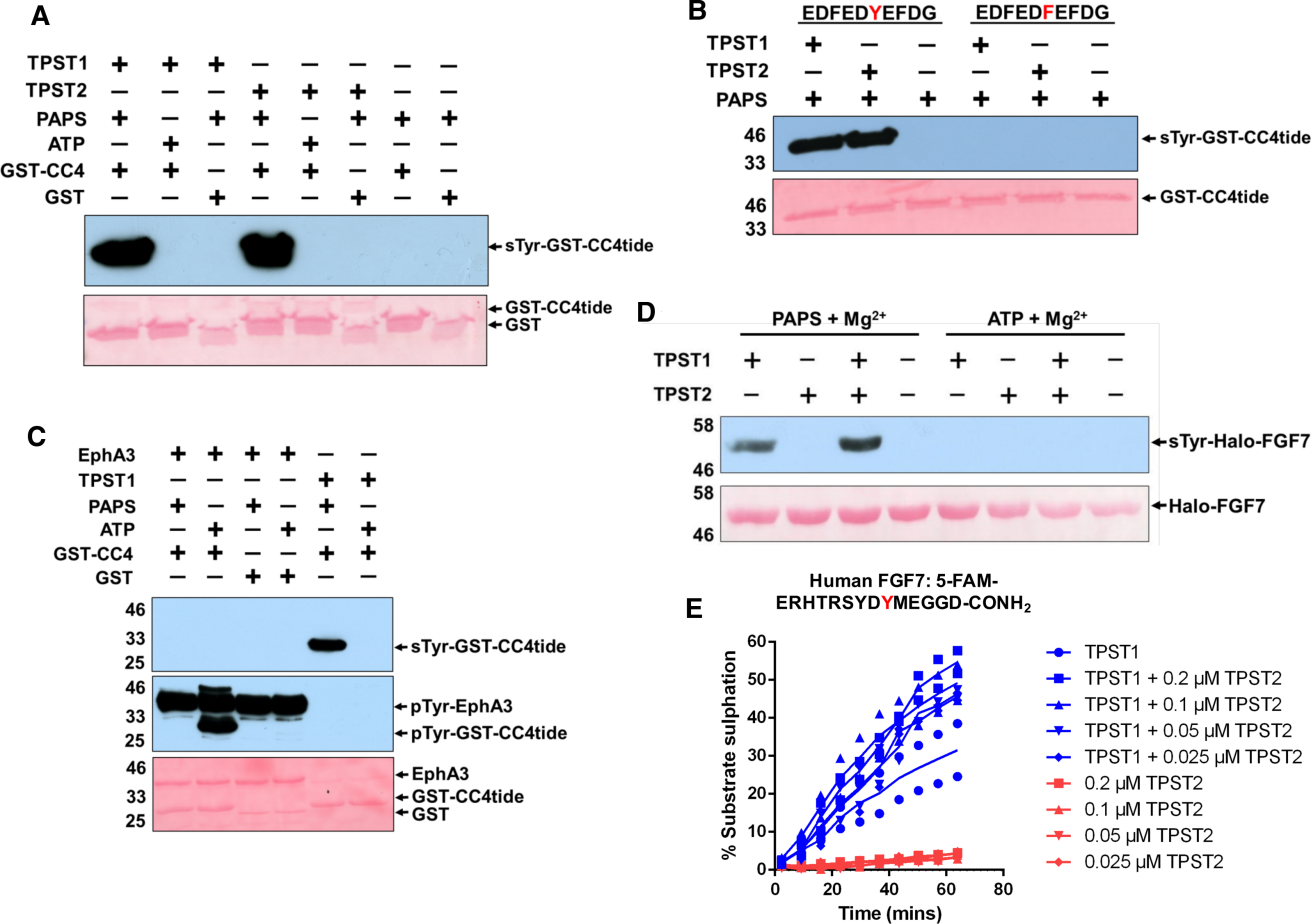
Validation and comparison of *in vitro* recombinant TPST sulfotransferase activities. (**A**) Immunoblot of an *in vitro* sulfotransferase assay using a recombinant GST-tagged CC4-tide. Recombinant, purified GST-CC4-tide, or purified GST alone (1 µg) was incubated at room temperature for 1 h with 1 µg of TPST1 or TPST2 ± 500 µM PAPS (sulfate donor) or 500 µM ATP (phosphate donor). (**B**) Immunoblot demonstrating TPST1- and TPST2-dependent sulfation of GST-CC4-tide at the specific tyrosine residue sulfated in intact CC4 (Tyr741). The assay was performed with 1 µg of each TPST enzyme, GST-CC4tide or GST-CC4-tide (Tyr741Phe), and 500 µM PAPS for 1 h at room temperature. (**C**) Western blot confirming that monoclonal sulfotyrosine antibody does not cross-react with phosphotyrosine. About 1 µg of GST-CC4-tide and GST was incubated for 1 h with 2 µg of TPST1 or EphA3 ± 500 µM PAPS or 500 µM ATP. GST CC4-tide sulfation (top panel), EphA3 autophosphorylation, or GST-CC4-tide phosphorylation (middle panel) is indicated. (**D**) Detection of *in vitro* recombinant FGF7 sulfation by immunoblot. Halo-tagged FGF7 (5 µg) was incubated for 16 h at 20°C with 2 µg of TPST protein ± 500 µM PAPS or 500 µM ATP, and tyrosine sulfation was detected using a monoclonal sulfotyrosine antibody (top panel). For all immunblotting-based assays, equal loading of substrate proteins was confirmed by Ponceau S staining (bottom panels). (**E**) To assess effects of TPST2 on TPST1 activity, TPST1 (fixed at 0.1 μM, blue symbols) was pre-incubated with the indicated concentration of TPST2 and assayed in the presence of 10 μM PAPS and 2 μM fluorescent FGF7 peptide. Peptide sulfation was monitored in real time by mobility analysis, and compared with control reactions lacking TPST2, or with indicated concentrations of TPST2 lacking TPST1 (red symbols).

Detection of quantitative tyrosine sulfation using real-time microfluidics represents a new approach to study this covalent modification *in vitro*. To unambiguously confirm sulfation by TPST1 and TPST2 using a complementary technique, we used immunoblotting with a monoclonal antibody that specifically recognises sulfated tyrosine in intact proteins. Initially, we generated a recombinant protein consisting of GST fused to the CC4-tide sequence (EDFED**Y**EFDG) that was developed for TPST1 and TPST2 mobility-based enzyme assays (Figures 2 and 3). As detailed in Figure 4A, this GST fusion protein became sulfated on tyrosine only after incubation with TPSTs, and this modification required PAPS in the assay. Consistently, GST was not detectably sulfated under any condition, confirming that the CC4-tide sequence was the target of both enzymes. Site specificity in the assay was confirmed by mutation of Tyr to Phe in the GST fusion protein, which abolished detection by the sulfotyrosine antibody (Figure 4B). As a further control, we demonstrated that GST-CC4-tide could become Tyr phosphorylated, but not Tyr sulfated, after incubation with Ephrin A3 and Mg-ATP at the same tyrosine sulfated by TPST1/2 in GST-CC4-tide (Figure 4C, note detection of pTyr in EphA3 protein due to autophosphorylation). This experiment also demonstrates unequivocally that the modification-specific antibodies can differentiate between sulfated and phosphorylated forms of the GST-CC4-tide protein. We also confirmed that full-length recombinant FGF7 was specifically modified by TPST1, but not by TPST2 *in vitro*, consistent with side-by-side kinetic analysis of TPST1 and TPST2 FGF7-tide sulfation, which also suggested a 2-fold enhancement of recombinant FGF7 sulfation when TPST1 and TPST2 were both included in the assay (Figures 2D and 4D). Given the known interaction between exogenously expressed TPST1 and TPST2 in the Golgi compartment [23], we evaluated this latter observation using a fluorescent FGF7 peptide, confirming that the rate and extent of peptide sulfation by TPST1 was enhanced in a dose-dependent manner by inclusion of TPST2 in the assay (Figure 4E).

### Analysis of TPST inhibition by biochemicals and the protein kinase inhibitor rottlerin

**Figure 5.**
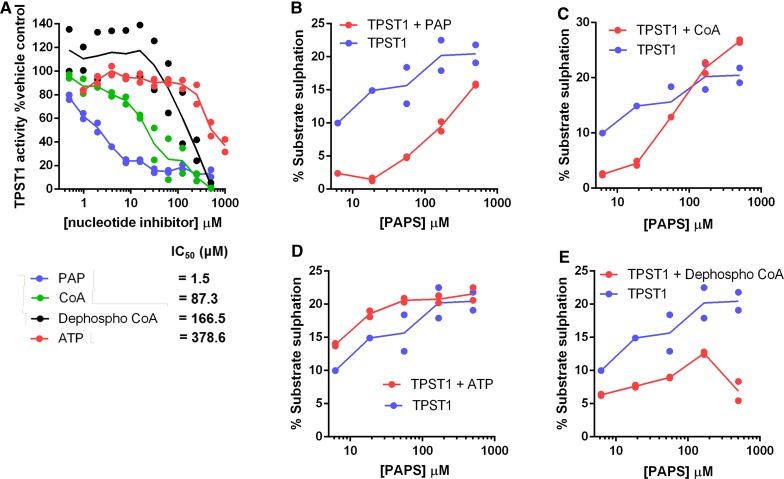
Nucleotide-dependent inhibition of TPST1 sulfotransferase activity varies with PAPS. (**A**) Dose–response curves and IC_50_ values for a panel of nucleotides incubated with TPST1 in the presence of 10 µM PAPS cofactor. TPST1 activity was measured using CC4-tide and normalised to controls containing buffer alone. (**B–E**) TPST1-dependent CC4-tide sulfation was measured in the presence of increasing PAPS concentration and a fixed concentration of (**B**) 20 µM PAP, (**C**) 100 µM ATP, (**D**) 100 µM CoA, or (**E**) 100 µM dephospho-CoA. All assays were performed using 0.1 µM TPST1 in the absence of MgCl_2_.

The ability of fluorescent peptide substrates to report TPST1- and TPST2-directed tyrosine sulfation in a plate-based assay format allowed us to develop an enzyme screen for the analysis and discovery of small-molecule TPST inhibitors. Based on the relative ease of purification and highly stable activity towards multiple substrates, we focused our biochemical screening studies on TPST1. As detailed in Figure 5A, the TPST ligands PAP, CoA, dephospho-CoA, and ATP were all able to inhibit PAPS-dependent sulfation of fluorescent CC4-tide by TPST1 *in vitro*. The IC_50_ values for inhibition ranged from low to high μM. This finding is consistent with the ability of PAP (Δ*T*_m_ = ∼10°C) and CoA (Δ*T*_m_ = ∼11°C), the two most potent inhibitors in the enzyme assay, to interact with and stabilise TPST1 (and TPST2) in thermal shift assays. Interestingly, the ability of PAP (IC_50_ = 1.5 μM) and CoA (IC_50_ = 87 μM) to inhibit TPST1 sulfation activity was highly sensitive to the concentration of PAPS in the assay, with peptide sulfation in the assay increasing (less inhibition) as a function of increasing PAPS, even taking into account the increases in enzyme activity induced by high levels of PAPS (Figure 5B,C). In contrast, weak TPST1 inhibition by ATP and dephospho-CoA was largely insensitive to an increase in PAPS levels in the assay, suggesting that it was likely to represent weak or non-competitive enzyme binding (Figure 5D,E).

**Figure 6.**
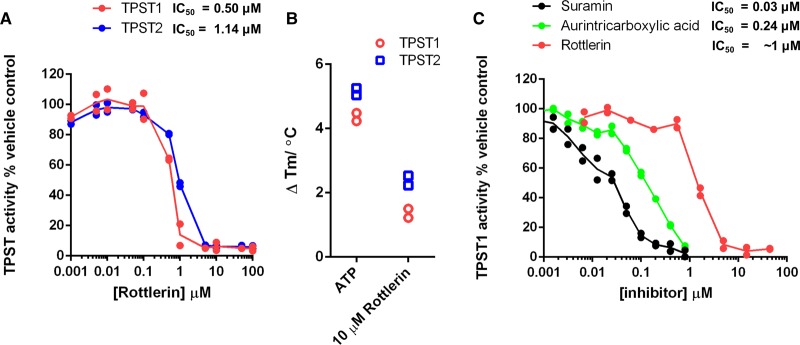
Targeting TPST sulfotransferase activity with small-molecule inhibitors. (**A**) Dose–response curves and calculated TPST IC_50_ values for rottlerin. TPST1 and 2 were incubated with the indicated concentration of rottlerin in the presence of 10 µM PAPS. TPST sulfotransferase activity towards CC4-tide was normalised to control reactions containing 1% (v/v) DMSO. (**B**) Thermal stability of purified TPST1 or TPST2 (5 µM) was measured in the presence of 10 µM rottlerin or 500 µM ATP as control. Δ*T*_m_ values were calculated by DSF as previously described. (**C**) TPST1 IC_50_ values for the previously described compounds suramin, aurintricarboxylic acid, and rottlerin were calculated using TPST1 and CC4-tide in the presence of 10 µM PAPS, and activity was normalised to control reactions containing 1% (v/v) DMSO.

Several literature reports suggest that TPST1/2 are inhibited by nucleotide and non-nucleotide compounds *in vitro* [35,59,60]. Using quantitative TPST1 and 2 enzyme assays, we identified that the broad-spectrum kinase inhibitor rottlerin, which was originally described as a ‘specific’ cellular PKC inhibitor [61], but later revealed to be a non-specific protein kinase inhibitor [62], inhibited PAPS-dependent TPST1 and TPST2 with single-digit micromolar IC_50_ values (Figure 6A). We also evaluated the clinically applied orphan compound suramin [63] and the DNA polymerase inhibitor aurintricarboxylic acid [64] as TPST inhibitors (Figure 6C), demonstrating inhibition with low-micromolar IC_50_ values, validating recent independent findings [35]. Consistently, we confirmed that rottlerin binding also induced a positive TPST1 and TPST2 thermal shift (Figure 6B), although the degree of stabilisation relative to ATP was lower than predicted, given the potent inhibitory effect of rottlerin on TPST1 and TPST2 enzyme activity *in vitro*.

### Multiple RAF kinase inhibitors target TPST catalytic activity *in vitro*

**Figure 7.**
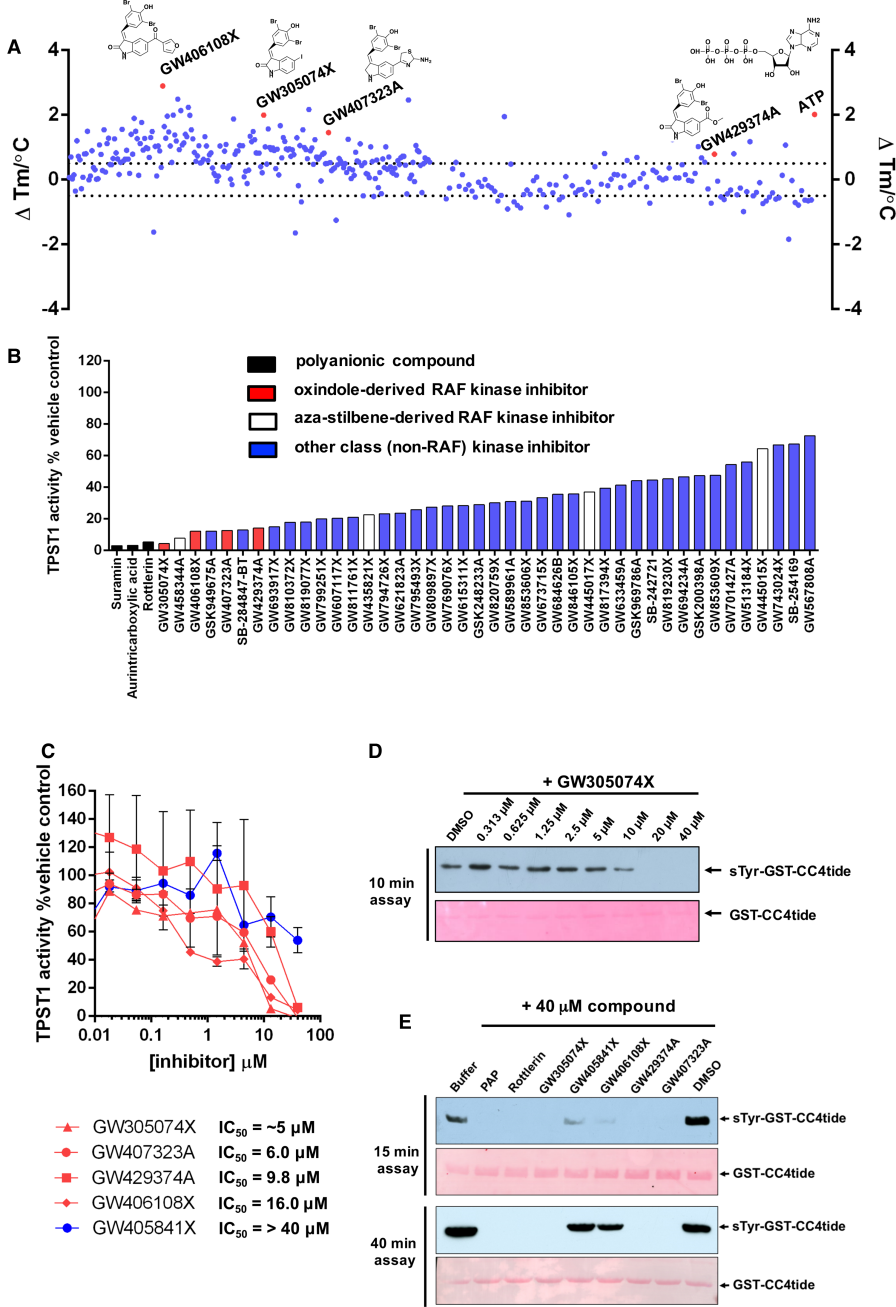
Mining the PKIS inhibitor library for TPST1 inhibitors. (**A**) Identification by DSF of PKIS small-molecule ligands that alter TPST1 thermal stability. TPST1 (5 µM) was screened using PKIS compounds at a final concentration of 20 µM compound and 4% (v/v) DMSO. Δ*T*_m_ values were calculated by subtracting the control *T*_m_ value (DMSO alone, no inhibitor) from *T*_m_ values. Data shown are a scatter plot of the mean Δ*T*_m_ values from two independent DSF-based assays. (**B**) Enzymatic inhibition of TPST1 sulfotransferase activity by selected PKIS compounds. TPST1 (0.1 µM) was incubated with the appropriate PKIS compound (40 µM) in the presence of 10 µM PAPS for 30 min at 37°C. TPST1 activity was measured using CC4-tide and normalised to 4% (v/v) DMSO control. The chemical class of inhibitor identified is colour-coded. (**C**) Compound dose–response and estimated IC_50_ values for selected chemical classes of PKIS inhibitors. TPST1 (0.1 µM) was incubated with increasing concentrations of the indicated inhibitor in the presence of 10 µM PAPS for 30 min at 37°C. TPST1 activity was measured using CC4-tide and normalised to DMSO controls. The data shown (and IC_50_ values) were calculated from duplicate experiments. (**D**) TPST1 (0.2 µg) was assayed using 1 µg of GST-CC4-tide and 10 µM PAPS in the presence of the indicated concentrations of GW305074X for 10 min prior to immunoblotting with monoclonal sulfotyrosine antibody Sulfo-1C-A2. (**E**) Immunoblots evaluating time dependence of TPST1 sulfotransferase activity in the presence of a panel of PKIS or several control inhibitors. GST-CC4-tide (1 µg) was incubated for the appropriate time in the presence of 0.2** **µg TPST1, 10 µM PAPS, and a fixed concentration (40 µM) of the indicated inhibitor. After reaction termination, tyrosine sulfation was subsequently visualised using monoclonal sulfotyrosine antibody (top panel), with equal GST-CC4-tide loading confirmed by Ponceau S staining (bottom panel). GST-CC4 sulfation was performed for either 15 min (top panels) or 40 min (bottom panels).

**Figure 8.**
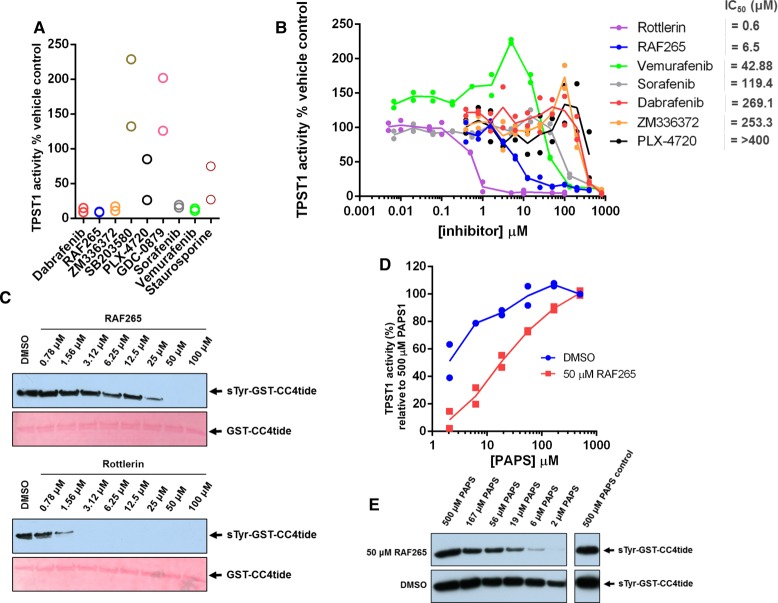
Evaluation of TPST1 inhibition by a panel of RAF kinase inhibitors. (**A**) Inhibition of TPST1 by RAF265 and other chemical classes of RAF inhibitor. TPST1 (0.1 µM) was pre-incubated with the appropriate inhibitor (400 µM) and the assay was initiated with PAPS (10 µM). (**B**) Dose–response and estimated IC_50_ values for TPST1 inhibition by RAF kinase inhibitors. TPST1 (0.1 µM) was pre-incubated with the indicated concentration of compound, and the assay was initiated with PAPS (10 µM). (**C**) Immunoblotting of GST-CC4-tide (1 µg) sulfation by TPST1 (1 µg) in the presence of increasing concentrations of RAF265 or rottlerin. TPST1 was pre-incubated with the indicated concentration of inhibitor, and assays were performed in the presence of 10 µM PAPS for 15 min at 20°C. (**D**) Antibody-based quantification of GST-CC4-tide sulfation by TPST1 in the presence of RAF-265 as a function of PAPS concentration. The tyrosine sulfation of GST-CC4-tide (a measure of TPST1 activity) was quantified by densitometry with the IMAGE J software. Data were normalised to sulfation in the presence of 500 µM PAPS and 4% (v/v) DMSO, which represents 100% activity in the absence of the inhibitor. (**E**) A representative immunoblot corresponding to the data quantified in (**D**) is presented.

The provocative chemical and structural similarities between CoA, PAP, ATP, and PAPS (Figure 1A), combined with the inhibitory effects of rottlerin on TPST1 and 2, raised questions about the general sensitivity of TPST enzymes to ATP-competitive kinase inhibitors. These findings prompted us to screen the open access PKIS, a collection of high-quality class-annotated kinase inhibitors assembled as a starting point to discover new chemical probes for enzyme targets. The commonality of the nucleotide-binding site in huge numbers of human proteins, and shared PAPS cofactor specificity in sulfotransferases made PKIS an attractive, unbiased resource for identifying potential new inhibitors for this family of enzymes. We took a dual-pronged approach for ligand screening, employing firstly a rapid TPST1 DSF assay and secondly a TPST1 enzyme assay. For DSF, a 20 μM compound concentration was employed for screening with 1 mM ATP as positive control, and we used a reproducible cut-off value of ∼Δ*T*_m_ ± 0.5°C in order to define a ‘hit’ (Figure 7A). The top compound found through this approach was GW406108X, and we noted strong thermal shifts in TPST1 by several compounds belonging to this indole-based kinase inhibitor class (red, Figure 7A and Supplementary Figure S4). Each ‘hit’ compound was next re-screened in a TPST1 enzyme sulfation assay at 40 μM, and the activity remaining compared with DMSO control (Figure 7B). Consistent with our DSF assay, five of the top seven TPST1 inhibitors were previously known RAF inhibitors with IC_50_ values for TPST1 in the low μM range, approximately an order of magnitude less potent than that of rottlerin (compare Figures 6C and 7C). These compounds were from two distinct types of previously described RAF inhibitors: derivatives of indole [65] or aza-stilbene [66] chemical classes. We next confirmed that GW305074X was a low-micromolar inhibitor of tyrosine sulfation by immunoblotting (Figure 7D), and investigated the rank order of potency for various indole compounds using GST-CC4-tide tyrosine sulfation, which was compared alongside rottlerin and PAP using the sulfotryosine-specific antibody (Figure 7E). Some limited structure–activity relationships emerged from these initial screens, prompting us to evaluate whether our data permitted us to predict generalised TPST inhibition by other RAF inhibitors, including clinically approved [67] and probe [68] compounds (Supplementary Figure S4). As shown in Figure 8A, duplicate assays revealed inhibition at a high (400 μM) concentration by many, but not all, RAF inhibitors tested, with dabrafenib, RAF-295, ZM336372, sorafenib, and vemurafenib showing essentially complete TPST1 inhibition at this concentration. The titration of each compound confirmed a complex profile of inhibition, with some RAF inhibitors (e.g. vemurafenib) potentially inducing partial TPST-1 activation at lower concentrations, and then inhibiting activity at higher concentrations (Figure 8B), perhaps consistent with their complex mode of interaction with RAF, which includes promotion of dimerisation and activation [69,70]. The most compelling inhibitory data were obtained with RAF265, a phase I imidazo-benzimidazole RAF inhibitor [71,72], for which an IC_50_ value of 6.5 μM towards TPST1 was measured, some 10-fold higher than that of rottlerin (Figure 8B). As shown in Figure 8C, both compounds exhibited dose-dependent inhibition when assayed in the presence of TPST1 and PAPS using GST-CC4-tide, and inhibition by RAF265 could be competitively decreased by increasing the concentration of PAPS in the assay, suggesting a partially competitive mode of inhibition with PAPS (Figure 8D,E).

### Docking analysis of TPST ligands

**Figure 9.**
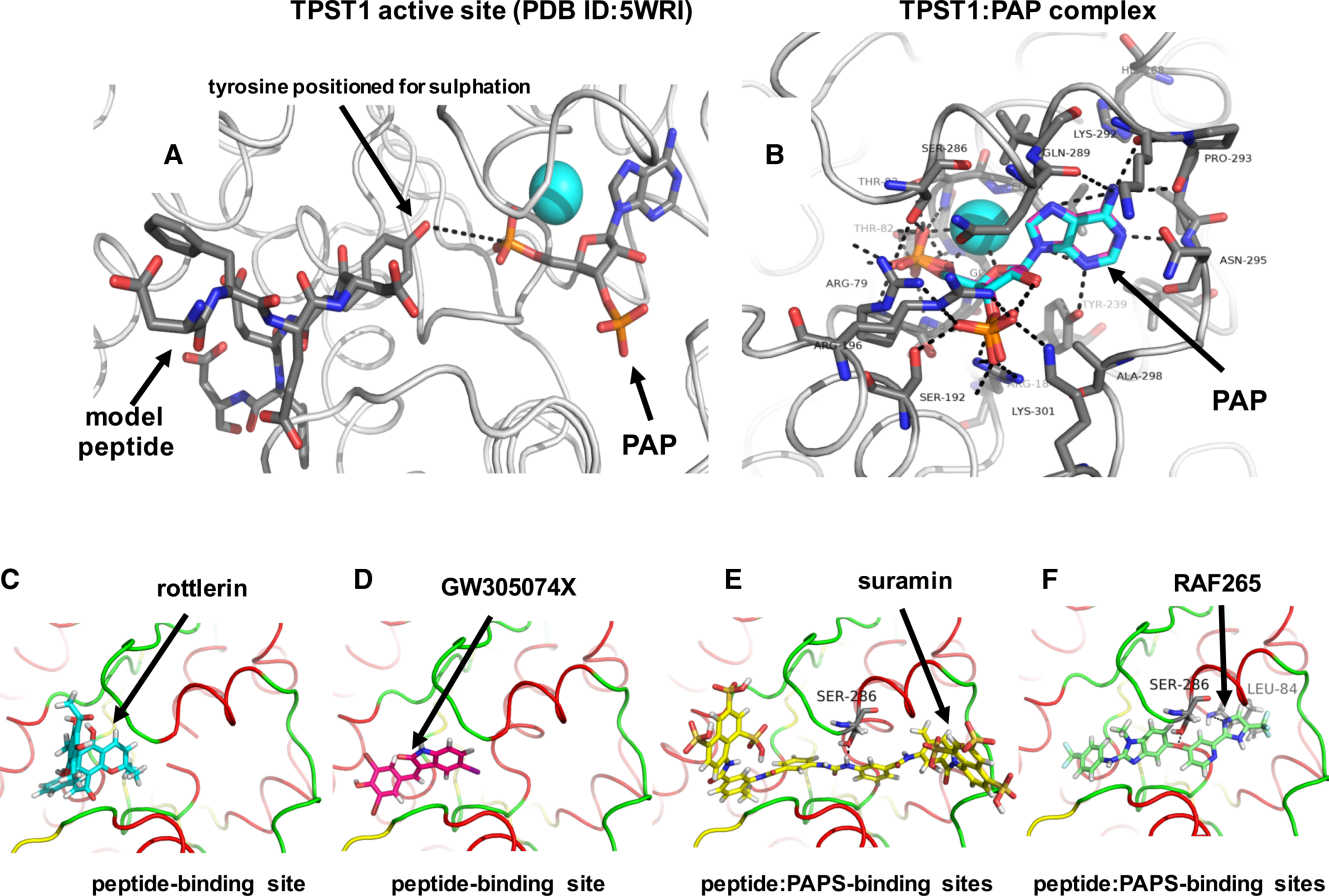
Molecular docking analysis of TPST1 with small-molecule inhibitor compounds. (**A**) Structure of human TPST1 complexed with adenosine-3′-5′-diphosphate (PAP) and the human CC4-derived substrate EDFED**Y**EFD PDB ID: 5WRI (protein rendered in grey cartoon). The inhibitory cofactor PAP (which replaces the physiological cofactor PAPS) and the co-crystallised CC4 substrate peptide are rendered as coloured sticks. Atoms are coloured grey (carbon), red (oxygen), blue (nitrogen), or cyan (oxygen of crystallographic water). Black dotted line indicates the close proximity of the tyrosyl hydroxyl group and PAP. (**B**) TPST1 docking poses compared. Experimentally derived (PDB ID: 5WRI) crystallographic carbons (cyan) or our modelled docking carbons (purple) are overlaid for the inhibitory cofactor mimic PAP. TPST1 was rendered as a cartoon. PAP shown in coloured sticks. Black dotted lines indicate hydrogen bonds. Rottlerin (**C**), GW305074X (**D**), suramin (**E**), or RAF265 (**F**) were all docked into human TPST1 (PDB ID: 5WRI), although docking solutions for each inhibitor could also be made with the very similar TPST2 catalytic domain (PDB ID: 3AP1). Proteins are depicted as cartoons with the following features: red — α-helix, yellow — β-sheet, green — loop. Docked molecules are coloured as sticks. Black dotted lines indicate potential hydrogen bonds.

To model the interaction of hit and control TPST1/2 ligands, including rottlerin, suramin, the sorafenib-derivative RAF265, and GW305074X with TPST1, we employed molecular docking to evaluate potential binding modes of compounds using the crystal structure of TPST1 (PDB ID: 5WRI). As shown in Figure 9A, like TPST2 [29], TPST1 possesses two adjacent docking sites in the extended catalytic region that accommodate binding of substrates, placing the tyrosine-containing substrate (left site) in proximity to the sulfate group of PAPS (right site). A docking protocol for the sulfation end-product PAP (adenosine-3′-5′-diphosphate) was developed that almost perfectly matched the crystallographic binding pose of this ligand for TPST1 (RMSD 0.30 Å, Figure 9B). By comparing experimentally favoured configurations with those of the crystallised ligands (PAP and a CC4 peptide poised for sulfation), we were able to confidently dock rottlerin, suramin, RAF265, and GW305074X into the TPST1 active site. These compounds are predicted to make many stabilising interactions that help explain their ability to act as inhibitors of TPSTs *in vitro* (Figure 9C–F). For example, rottlerin (C) and GW305074X (D) are predicted to occupy the peptide-binding site of TPST1, while suramin (E) and RAF265 (F) span both the peptide and PAPS-binding sites, consistent with the competitive loss of TPST1 inhibition by RAF265 observed as the concentration of PAPS increases in enzyme assays (Figure 8D). In contrast, suramin is predicted to form a hydrogen bond with Ser286, while RAF-265 forms hydrogen bonds with both Ser286 and Leu84.

## Discussion

TPSTs catalyse protein sulfation using PAPS as the sulfate group donor, and are thought to possess structural [29] and biochemical similarities with protein tyrosine kinases relevant to both binding of synthetic substrates and an ability to modify them enzymatically *in vitro* [73]. Overlapping sulfate or phosphate modifications can potentially occur on the same tyrosine residue when appropriate acidic residues dock the substrate into the active site for covalent modification [26,74]. Although the physiological relevance of combinatorial and competitive tyrosine modification on phosphate or sulfate (or nitrate) remains essentially unknown, bioinformatic analysis incorporating secondary structural analysis predicts that >20 000 context-dependent protein tyrosine residues are sulfated in the human proteome [30,31,75]. However, due to a lack of chemical tool compounds, sulfation is understudied in living organisms, often relying on ‘sledgehammer’ approaches employing non-specific reagents such as chlorate or total genetic ablation [36]. The analysis of tyrosine sulfation remains ripe for both technological innovation and the discovery of new classes of sulfotransferase inhibitor [76], in order to promote new chemical biology approaches in the field.

### DSF and sulfotransfer analysis of TPST1 and TPST2

In the present paper, we report a simple and rapid method for the detection of TPST-catalysed peptide sulfation using model substrates fused to an N-terminal fluorophore. The chemical similarity between the phosphate donor ATP and PAPS, the universal sulfate donor, led us to investigate whether peptide tyrosyl sulfation could be detected using a high-throughput enzymatic procedure previously validated for phosphorylation catalysed by ATP-dependent kinases. To isolate pure, enzymatically active recombinant TPST1 and TPST2, both were expressed at high levels in bacteria, and refolded after purification from inclusion bodies using published ‘slow’ procedures suitable for structural and enzymatic analysis of TPST1 [24] and TPST2 [29]. The affinity of our TPST1 and TPST2 preparations for the PAPS cofactor was found to be almost identical with that previously reported, and we confirmed that TPST1 and TPST2 were folded and could bind to a variety of physiological and non-physiological ligands. These included sulfated PAPS and PAP, the end product of the sulfotransferase reaction (Figure 1 and Supplementary Figure S1). Protein kinases are also known to bind to the reaction end product of the phosphotransferase reaction (ADP), which can act as a weak ATP-competitive inhibitor [48]. Our study also revealed that TPST1 and 2 interact with the 3′-phospho-adenosine moiety of the ligand CoA, confirming the availability of the 3′-phospho-adenosine docking region in the active site of TPSTs for unrelated ligand binding. To our knowledge, our studies are the first to employ DSF-based thermal shift assays to analyse TPST ligand binding, although these approaches are also widely used for semi-quantitative ligand-binding analysis of growth factors [45,77], protein kinase [48,55–57] and pseudokinase [78] domains, BH3 domains [47] and bromodomains [79].

Standard biochemical assays often involve the detection of ^35^S-based substrate sulfation derived from ^35^S-labelled PAPS, and require enzymatic cofactor synthesis and time-consuming radioactive solid-phase chromatography (typically HPLC) procedures [80,81]. In contrast, our peptide sulfation assay detects modification in real time using a simple mobility shift assay, which is quantified by comparing the ratio of the sulfated and non-sulfated fluorescent substrates. This assay employs the EZ-Reader II platform originally developed for the rapid analysis of peptide phosphorylation, acetylation, or proteolysis [50], and permits the inclusion of high concentrations of non-radioactive cofactors, substrates, and ligands. The coupling of a fluorophore at the peptide N-terminus, distinct from the site of tyrosine sulfation (Figure 2A), overcomes current limitations with fluorescent TPST substrates, in which the fluorophore lies adjacent to the site of sulfation. In the course of our studies, we established a high reproducibility for this assay and exploited it to probe substrate specificity and discover new enzyme inhibitors. We also generated a substrate lacking a key Tyr residue, a dual protein kinase/TPST substrate and model TPST substrates containing acidic residues at the −1 and +1 position relative to the sulfated Tyr. These allowed us to generate CCR8 and FGF7 substrates for TPST1, which were not substrates for TPST2, and dual substrates with differential (CC4-tide) or very similar (PSGL1) sulfation kinetics for TPST1 and TPST2. Based on our initial analyses, we found that TPST2 only sulfated tyrosine-containing peptide substrates with an acidic residue in both the +1 and −1 position, whereas TPST1-dependent tyrosine sulfation only required a negative charge to be present in the −1 site (Figure 2). Future work will employ a much larger selection of peptide substrates to evaluate this preference further, with a goal of defining TPST1 and TPST2 substrate specificity *in vitro* that can be exploited to help examine the sulfoproteomic datasets emerging from cell-based studies.

### New small-molecule TPST1 inhibitors

We confirmed by real-time analysis that TPST ligands act as competitive active-site inhibitors of peptide sulfation, creating a new impetus to develop novel screening approaches to discover TPST inhibitors. Our finding that TPST1 was inhibited at sub-micromolar concentrations by the anti-angiogenic compound suramin, which has been used clinically to treat River Blindness and African trypanasomiasis [63], and the cellular DNA polymerase inhibitor aurintricarboxylic acid [64] was intriguing, and consistent with a very recent report demonstrating inhibitory activity towards TPSTs [35]. By screening a panel of kinase inhibitors, we found that rottlerin (also known as mallotoxin) is also a low-micromolar inhibitor of TPST1 and TPST2 *in vitro*. Rottlerin was originally identified as an inhibitor of PKC isozymes [61], but can also act as a sub-micromolar inhibitor of other protein kinases *in vitro* [62]. Interestingly, we discovered that all three of these compounds also have inhibitory activity towards the related PAPS-dependent heparin sulfate 2-*O* sulfotransferase HS2ST *in vitro* [43], allowing us to infer that structural similarities in the PAPS or substrate-binding regions of HS2ST and TPST1/2 present a binding surface that accommodates small polyanionic compounds, which, like TPST acidic peptide substrates, presumably bind through electrostatic interactions in the enzyme active site. These findings heightened the possibility that other kinase inhibitors might also be serendipitous TPST inhibitors.

To evaluate this hypothesis, we identified many TPST1 ligands in PKIS. Intriguingly, four of the top seven hits in this screen belonged to the same benzylidene-1H-inol-2-one (oxindole) c-RAF kinase inhibitor subclass [65]. Moreover, of the other top 30 TPST inhibitors identified (TPST1 enzyme inhibition >40% at 20 μM), GW445015X, GW445017X, and most notably GW458344A, were all potent c-RAF inhibitors, belonging to the chemically distinct aza-stilbene chemical class [66]. We next confirmed that distinct RAF inhibitors also possess inhibitory properties towards TPST1. Interestingly, well-validated clinical RAF inhibitors, including RAF265 (IC_50_ 6.5 μM), vemurafenib (IC_50_ ∼40 μM), and the much higher micromolar TPST inhibitor sorafenib (which contains the same 2-arylaminobenzimidazole chemical scaffold found in RAF265, Supplementary Figure S4), were also TPST1 inhibitors *in vitro*. These findings demonstrate that many compounds designed as RAF inhibitors also have the ability to inhibit TPST1, providing a new impetus to exploit the huge amount of RAF inhibitor design knowledge available in private and public databases for the design and testing of TPST inhibitors. In a related paper, we demonstrated cross-reactivity of rottlerin- and oxindole-based (but not aza-stilbene or other RAF) inhibitors with the glycan sulfotransferases HS2ST [43]. Interestingly, the potency of HS2ST inhibition by oxindole-based c-RAF inhibitors was some 10-fold lower than that for TPST1, and we confirmed that TPST RAF inhibitors such as RAF265 and aza-stilbenes did not inhibit HS2ST at any concentration tested. These subtle differences suggest that although inhibitor sensitivity to this class of RAF inhibitors can be shared between two distinct classes of sulfotransferase, opportunities exist for the development of both specific and potent ligands targeted more specifically towards either HS2ST or TPSTs.

## Conclusions

To stimulate progress in implementing chemical biology in the sulfotransferase field, careful structure-based comparison between HS2ST, TPST1/2, and RAF kinase inhibition, and analysis of a wide variety of compound chemotypes obtained from high-throughput analysis, will be required. Our docking studies with TPST1 suggest similar binding modes for both rottlerin and the oxindole TPST1 inhibitor GW305074X (Figure 9), while suramin and RAF265 might feasibly interact with the extended peptide and PAPS cofactor-binding sites. It will be intriguing to confirm these binding modes through biophysical analysis and structure-guided enzyme mutagenesis, and to identify drug-binding site residues in sulfotransferases that dictate inhibition. This information can then be used for careful compound analysis and the generation of drug-resistant alleles for cellular analysis, using concepts developed for compound target validation in the kinase field [82–86]. In the first instance, it will also be important to evaluate whether any of the TPST ligands identified here, particularly cellular RAF inhibitors, interfere with protein tyrosine sulfation in cells, since it remains formally possible that some of the cellular phenotypes and/or clinical effects documented with these compound classes [67] might be explained in part by ‘off-target’ effects on sulfation-based biology. Cellular inhibition by such non-optimised compounds will probably depend on several unknown factors, most notably the cellular concentration of PAPS, the rate of tyrosine sulfation and desulfation, and the relative penetrance of compounds into the Golgi, where modification is thought to occur.

Our work also raises the possibility that TPST inhibitors might be synthesised or repurposed based on workflows previously developed for the iteration of the different families of (RAF) kinase inhibitors. Although only two TPSTs are present in multicellular eukaryotes, the development of specific inhibitors might be challenging, given the ∼90% similarity within the active site, the presence of multiple distinct PAPS-dependent sulfotransferases in vertebrate genomes, and the presence of endogenous competing PAPS present in the cell. However, in this context, it is useful to recall the rapid development of the kinase inhibitor field as an exemplar. Initial scepticism about the feasibility (or, even, the need) to generate specific inhibitors of protein kinases has been largely overcome [87], partly through innovative synthetic approaches, but also by a deep understanding of mechanistic and structural kinase biology available within the >500 distinct members of the human kinome [38]. An appreciation that compound polypharmacology, perhaps across multiple enzyme classes, is important for driving, and predicting, both efficacy and compound side effects for kinase inhibitors [88–90] might also be useful for the development of TPST inhibitors.

Finally, inhibitor-based interrogation of TPST-dependent tyrosine sulfation could be employed alongside MS-based sulfoproteomics [32,91]. This could have a significant impact in various areas of research by increasing our ability to chemically control and modulate tyrosine sulfation and even manipulate sulfation of specific proteins, including those implicated in, for example, infection and inflammation. Given the close parallels between tyrosine sulfation and tyrosine phosphorylation, whose rational targeting rapidly led to the analysis of large numbers of clinically relevant small-molecule inhibitors [87], we suggest that a new opportunity might also soon exist to integrate the analysis of TPST with the tools of chemical biology.

## Abbreviations

CCR8: C–C motif chemokine receptor 8
CoA: coenzyme A
DSF: differential scanning fluorimetry
FGF7: fibroblast growth factor 7
MS: mass spectrometry
PAP: 3′-phosphoadenosine 5′-phosphate
PAPS: 3′-phosphoadenosine 5′-phosphosulfate
PKIS: Published Kinase Inhibitor Set
PSGL-1: P-selectin glycoprotein ligand-1
RAF: rapidly accelerated fibrosarcoma
SEC: size-exclusion chromatography
TPST: tyrosylprotein sulfotransferase
TSA: thermal stability assay
