# Supplementary material for: New tools for evaluating protein tyrosine sulfation: tyrosylprotein sulfotransferases (TPSTs) are novel targets for RAF protein kinase inhibitors
Source: Biochem J. 2018 Aug 14;475(15):2435–55. doi: 10.1042/BCJ20180266 (PMC6094398; doi:10.1042/BCJ20180266)

Supplementary Figures

Supplementary Figure 1. DSF-based analysis of TPST1 and TPST2 ligand interactions.

Thermal stability profiles of **(A)** TPST1 or **(B)** TPST2 were measured in the presence of the indicated nucleotide. ΔT_m values were calculated relative to buffer controls for each TPST enzyme (5 μM) in the presence of 0.5 mM of the indicated nucleotide ± 10 mM $MgCl_2$.

Supplementary Figure 2. K_m [PAPS] determination for TPST1.

Kinetic analysis of CC4-tide (2 μM) sulphation by purified TPST1 (0.1 μM) was performed in the presence of increasing concentrations of the sulphate donor PAPS**.** The K_m [PAPS] value (± standard deviation) was calculated by comparing the rate of peptide sulphation (pmoles sulphate/min) and linear regression software (GraphPad Prism) from four independent experiments.

Supplementary Figure 3. Analysis of TPST1 and TPST2 activity in the presence of a panel of peptide substrates and selected divalent metal cations.

(A) The rate and extent of CC4-tide sulphation was measured as a function of Mg^{2+} or Mn^{2+} ion concentration. TPST1 or TPST2 (0.1 μM) were incubated with increasing concentrations of Mg^{2+} or Mn^{2+} in the presence of 10 μM PAPS. TPST1 activity was normalised to a buffer control. **(B-D)** Rate and extent of tyrosine sulphation of fluorescently-labelled tyrosine-containing substrate peptides derived from human FGF7 (B), CCR4 (C) or PSGL1 (D). Assays were conducted in the presence and absence of 10 mM Mg^{2+} or Mn^{2+} ions. Assays were performed at 20°C using 2 μM of the appropriate peptide, 10 μM PAPS and 0.1 μM TPST1 (red) or TPST2 (blue).

Supplementary Figure 4. Chemical structures of TPST ligands.

The chemical structures of rottlerin, suramin and aurintricarboxylic acid, a panel of TPST inhibitors discovered from PKIS and various known RAF inhibitors.

Figure 1

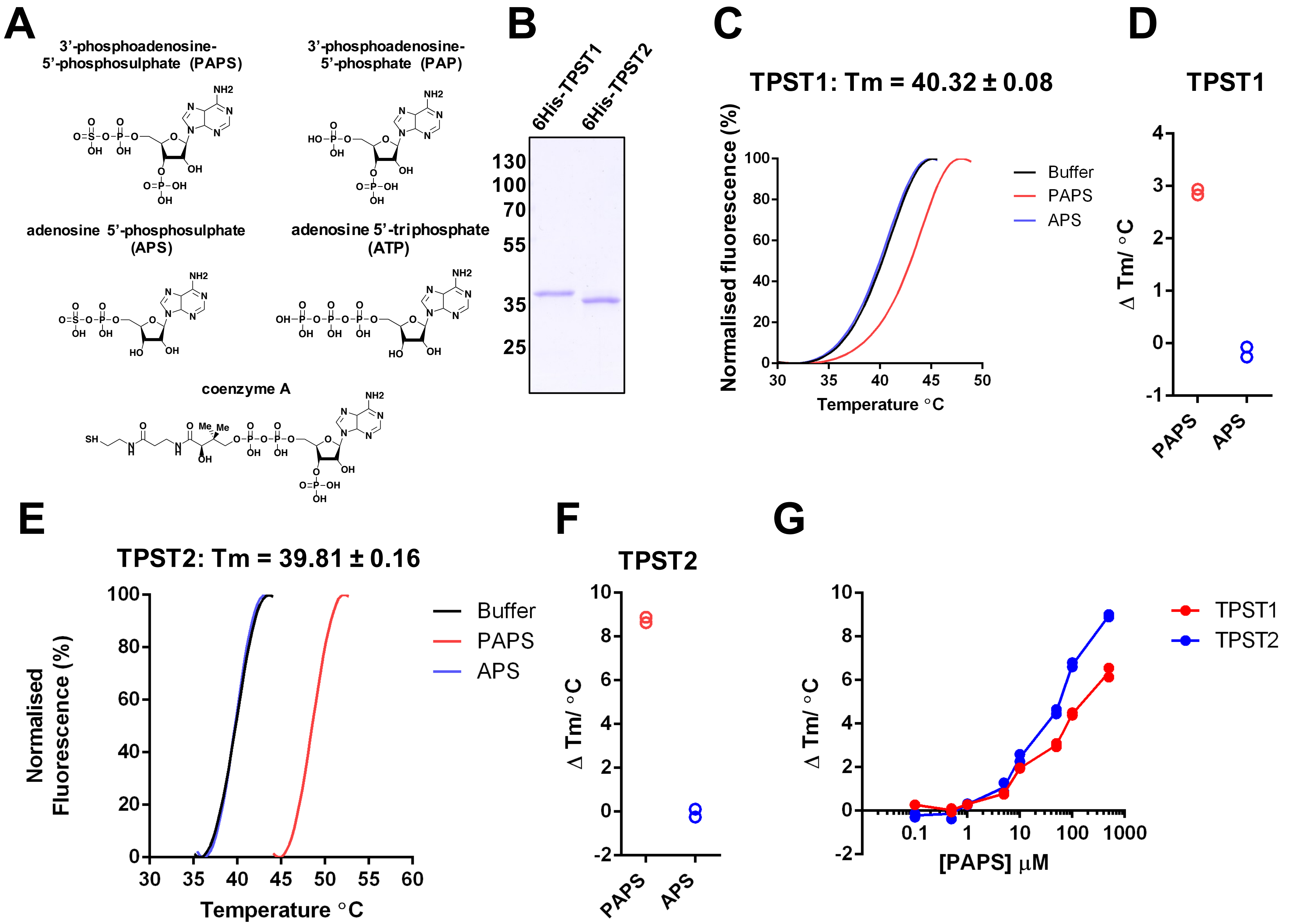

A
3'-phosphoadenosine-5'-phosphosulphate (PAPS)
3'-phosphoadenosine-5'-phosphate (PAP)
adenosine 5'-phosphosulphate (APS)
adenosine 5'-triphosphate (ATP)
coenzyme A
B
6His-TPST1
6His-TPST2
130
100
70
55
35
25
C
TPST1: Tm = 40.32 ± 0.08
Normalised fluorescence (%)
Temperature °C
Buffer
PAPS
APS
D
TPST1
Δ Tm/ °C
PAPS
APS
E
TPST2: Tm = 39.81 ± 0.16
Normalised Fluorescence (%)
Temperature °C
Buffer
PAPS
APS
F
TPST2
Δ Tm/ °C
PAPS
APS
G
Δ Tm/ °C
[PAPS] μM
TPST1
TPST2

Figure 2

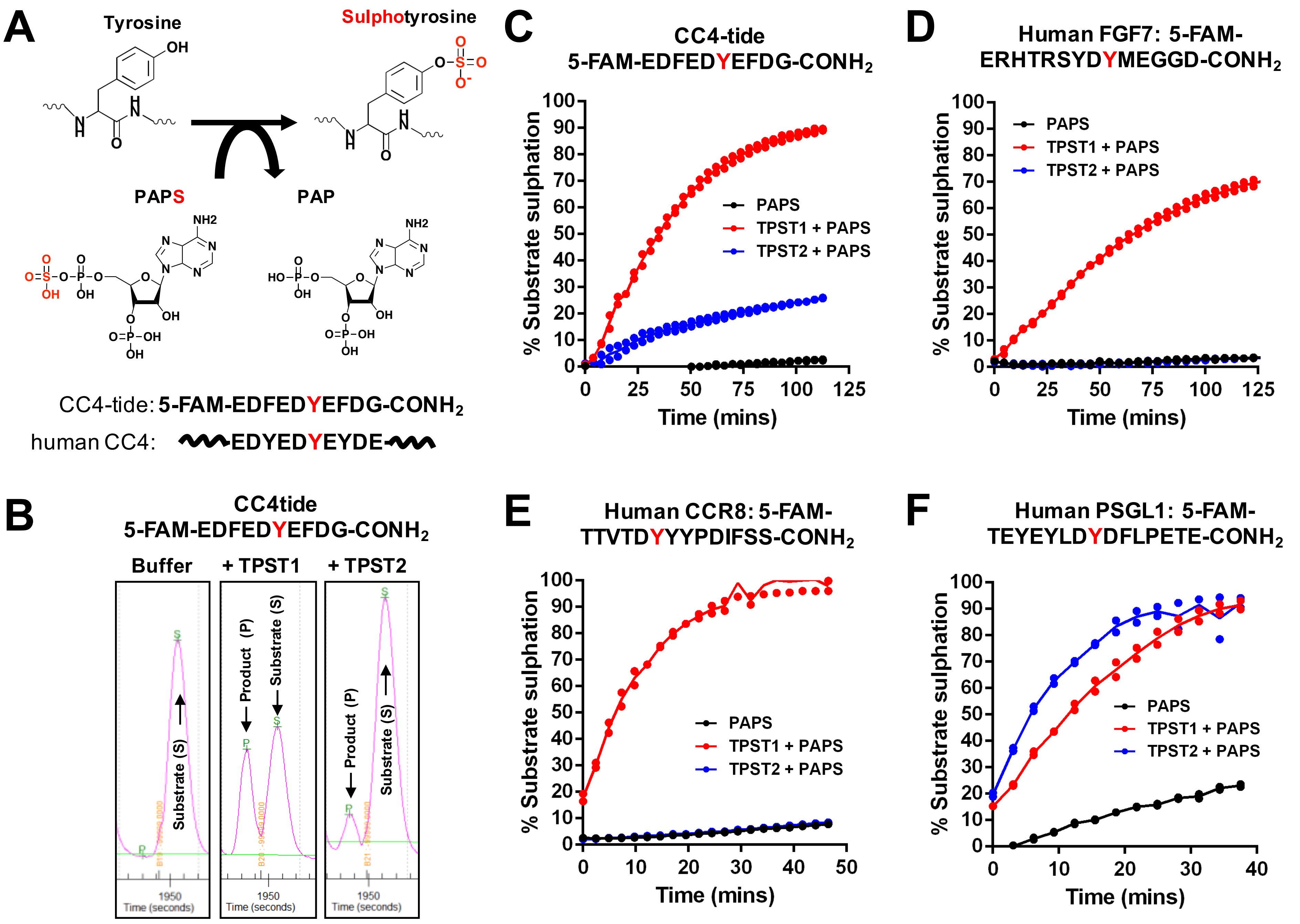
A
Tyrosine
Sulphotyrosine
PAPS
PAP
CC4-tide: 5-FAM-EDFEDYEFDG-CONH2
human CC4: EDYEDYEYDE
B
CC4tide
5-FAM-EDFEDYEFDG-CONH2
Buffer
+ TPST1
+ TPST2
Substrate (S)
Product (P)
Time (seconds)
C
CC4-tide
5-FAM-EDFEDYEFDG-CONH2
% Substrate sulphation
Time (mins)
PAPS
TPST1 + PAPS
TPST2 + PAPS
D
Human FGF7: 5-FAM-
ERHTRSYDYMEGGD-CONH2
E
Human CCR8: 5-FAM-
TTVTDYYYPDIFSS-CONH2
F
Human PSGL1: 5-FAM-
TEYEYLDYDFLPETE-CONH2

Figure 3

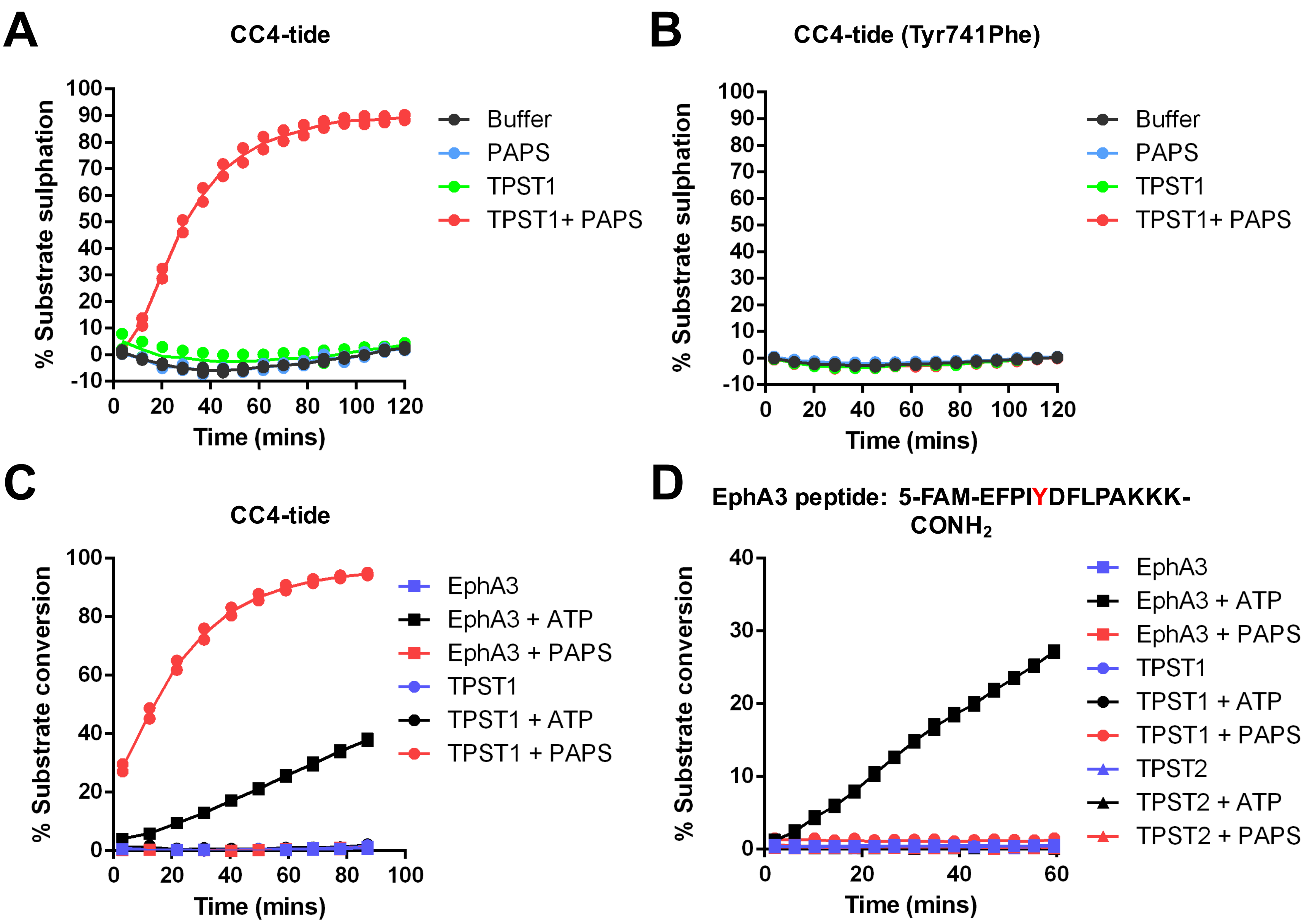

A
CC4-tide
% Substrate sulphation
Time (mins)
Buffer
PAPS
TPST1
TPST1+ PAPS
B
CC4-tide (Tyr741Phe)
% Substrate sulphation
Time (mins)
Buffer
PAPS
TPST1
TPST1+ PAPS
C
CC4-tide
% Substrate conversion
Time (mins)
EphA3
EphA3 + ATP
EphA3 + PAPS
TPST1
TPST1 + ATP
TPST1 + PAPS
D
EphA3 peptide: 5-FAM-EFPIYDFLPAKKK-CONH2
% Substrate conversion
Time (mins)
EphA3
EphA3 + ATP
EphA3 + PAPS
TPST1
TPST1 + ATP
TPST1 + PAPS
TPST2
TPST2 + ATP
TPST2 + PAPS

Figure 4

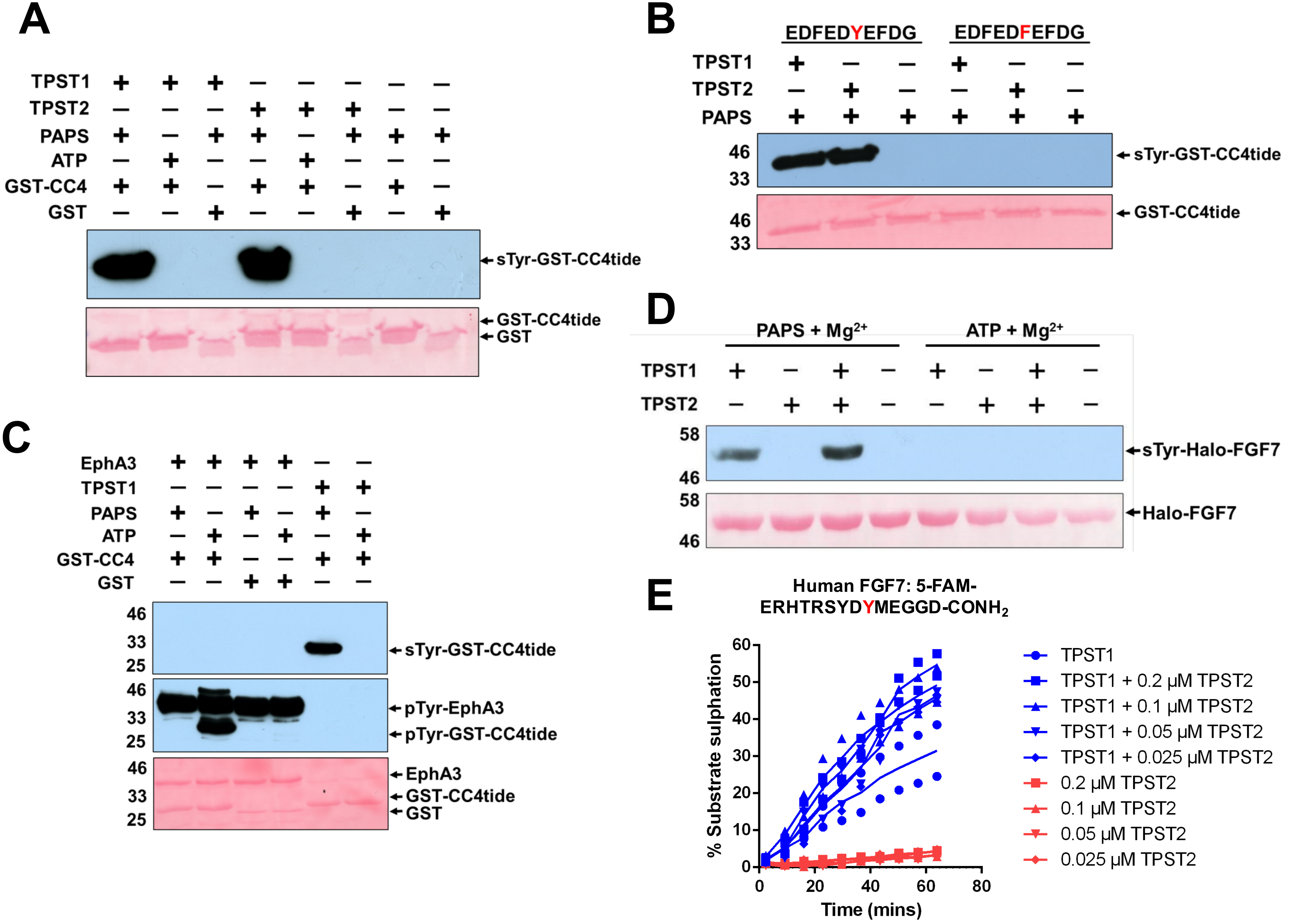

A
TPST1
TPST2
PAPS
ATP
GST-CC4
GST
sTyr-GST-CC4tide
GST-CC4tide
GST
B
EDFEDYEFDG
EDFEDFEFDG
TPST1
TPST2
PAPS
46
33
sTyr-GST-CC4tide
46
33
GST-CC4tide
C
EphA3
TPST1
PAPS
ATP
GST-CC4
GST
46
33
25
sTyr-GST-CC4tide
46
33
25
pTyr-EphA3
pTyr-GST-CC4tide
46
33
25
EphA3
GST-CC4tide
GST
D
PAPS + Mg2+
ATP + Mg2+
TPST1
TPST2
58
46
sTyr-Halo-FGF7
58
46
Halo-FGF7
E
Human FGF7: 5-FAM-ERHTRSYDYMEGGD-CONH2
% Substrate sulphation
Time (mins)
0
10
20
30
40
50
60
0
20
40
60
80
TPST1
TPST1 + 0.2 μM TPST2
TPST1 + 0.1 μM TPST2
TPST1 + 0.05 μM TPST2
TPST1 + 0.025 μM TPST2
0.2 μM TPST2
0.1 μM TPST2
0.05 μM TPST2
0.025 μM TPST2

Figure 5

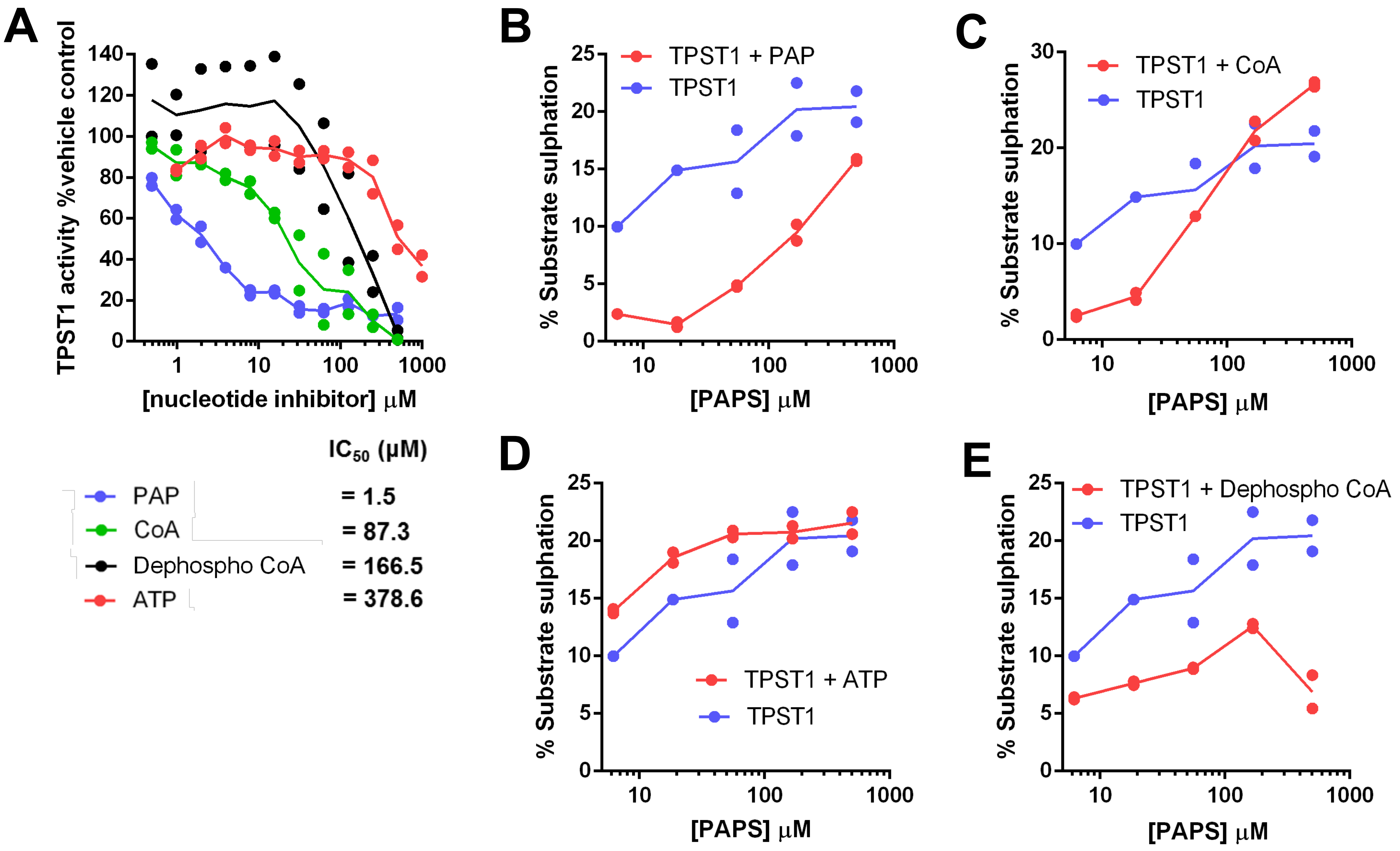
A
TPST1 activity %vehicle control
140
120
100
80
60
40
20
0
1
10
100
1000
[nucleotide inhibitor] µM
IC50 (µM)
PAP = 1.5
CoA = 87.3
Dephospho CoA = 166.5
ATP = 378.6
B
% Substrate sulphation
TPST1 + PAP
TPST1
[PAPS] µM
C
TPST1 + CoA
TPST1
D
TPST1 + ATP
TPST1
E
TPST1 + Dephospho CoA
TPST1

Figure 6

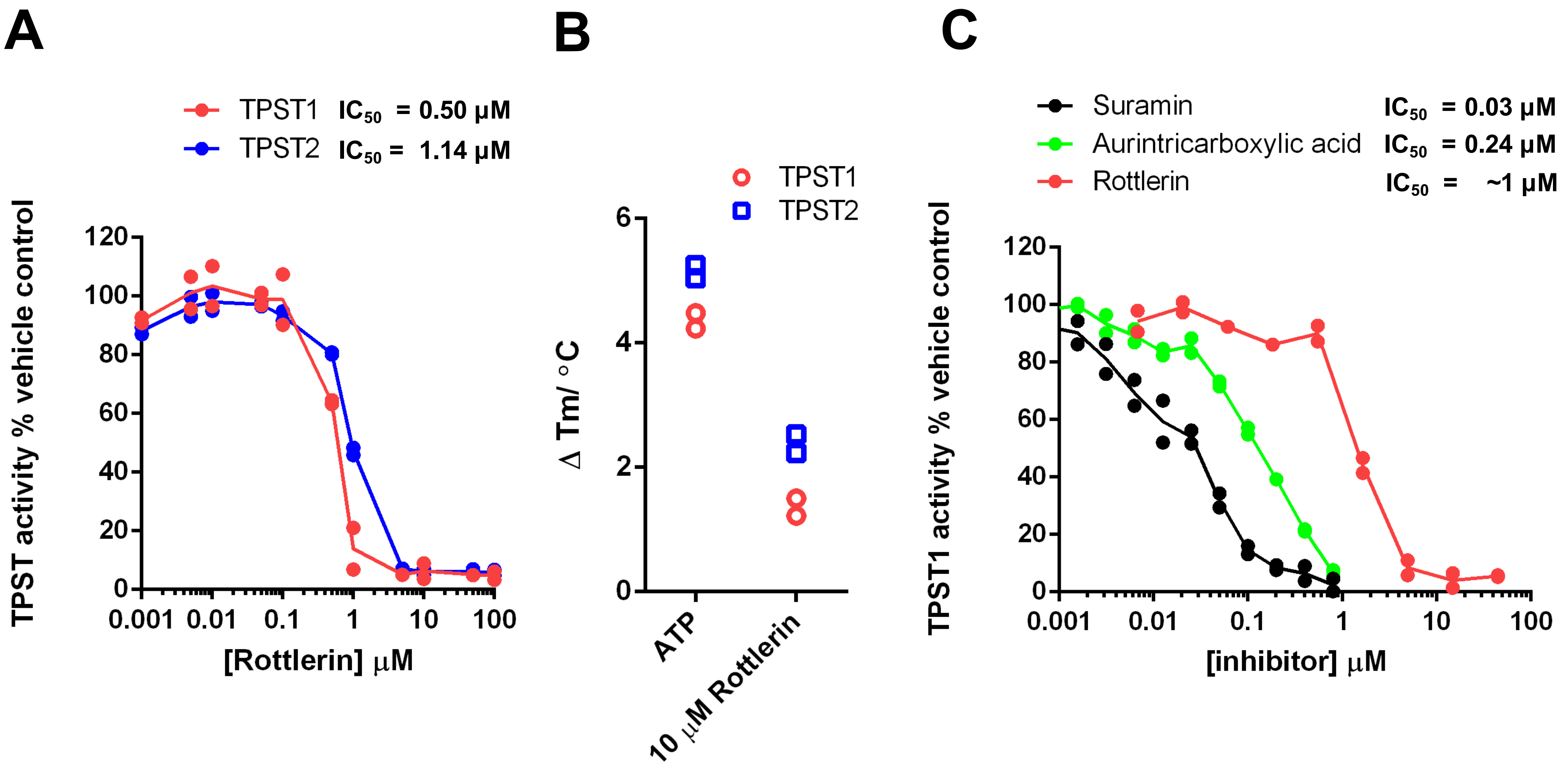
A
TPST1 IC50 = 0.50 μM
TPST2 IC50 = 1.14 μM
TPST activity % vehicle control
[Rottlerin] μM
B
TPST1
TPST2
Δ Tm/ °C
ATP
10 μM Rottlerin
C
Suramin IC50 = 0.03 μM
Aurintricarboxylic acid IC50 = 0.24 μM
Rottlerin IC50 = ~1 μM
TPST1 activity % vehicle control
[inhibitor] μM

Figure 7

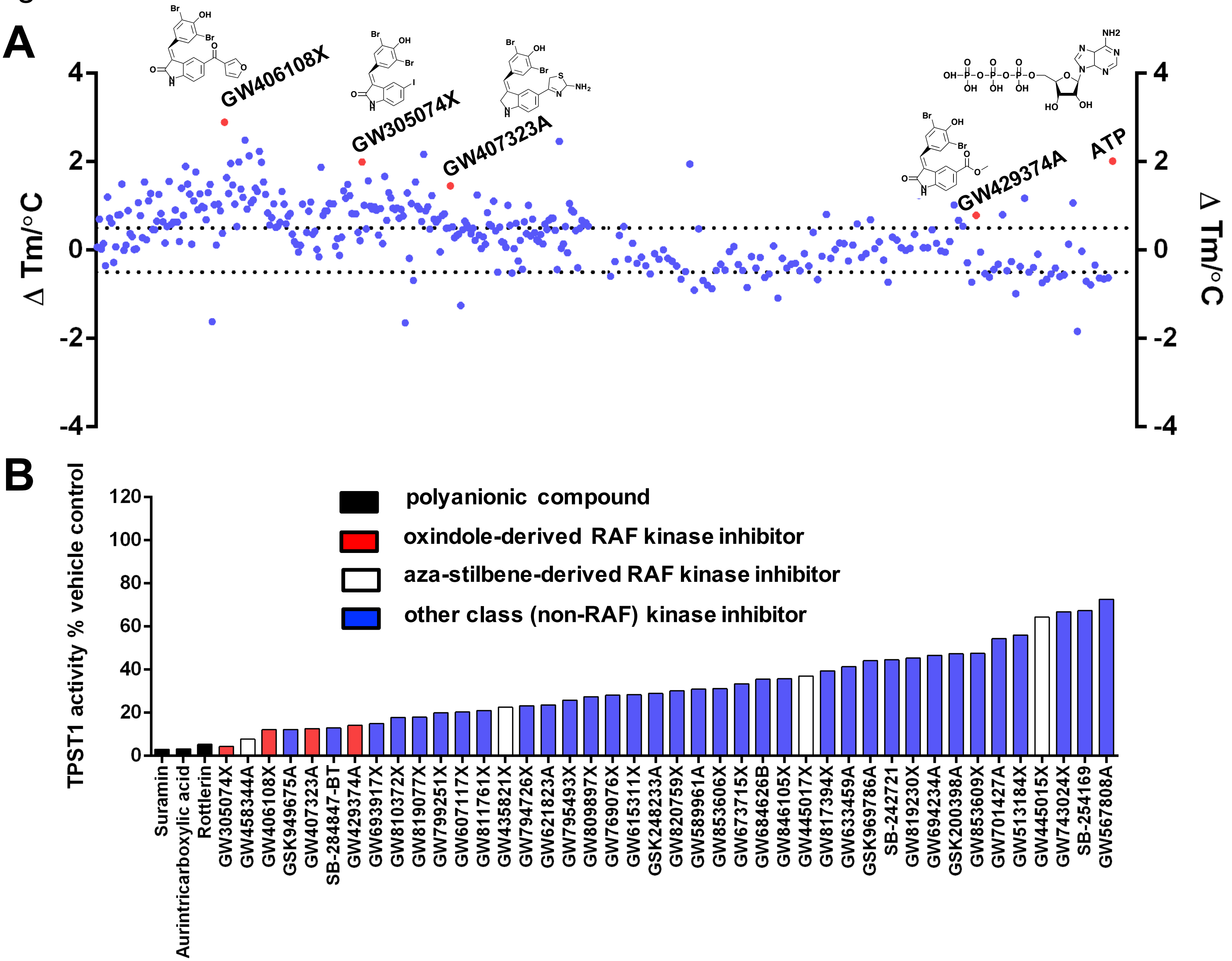
A
B
GW406108X
GW305074X
GW407323A
GW429374A
ATP
Δ Tm/°C
TPST1 activity % vehicle control
polyanionic compound
oxindole-derived RAF kinase inhibitor
aza-stilbene-derived RAF kinase inhibitor
other class (non-RAF) kinase inhibitor
Suramin
Aurintricarboxylic acid
Rottlerin
GW305074X
GW458344A
GW406108X
GSK949675A
GW407323A
SB-284847-BT
GW429374A
GW693917X
GW810372X
GW819077X
GW799251X
GW607117X
GW811761X
GW435821X
GW794726X
GW621823A
GW795493X
GW809897X
GW769076X
GW615311X
GSK248233A
GW820759X
GW589961A
GW853606X
GW673715X
GW684626B
GW846105X
GW445017X
GW817394X
GW633459A
GSK969786A
SB-242721
GW819230X
GW694234A
GSK200398A
GW853609X
GW701427A
GW513184X
GW445015X
GW743024X
SB-254169
GW567808A

Figure 7 continued

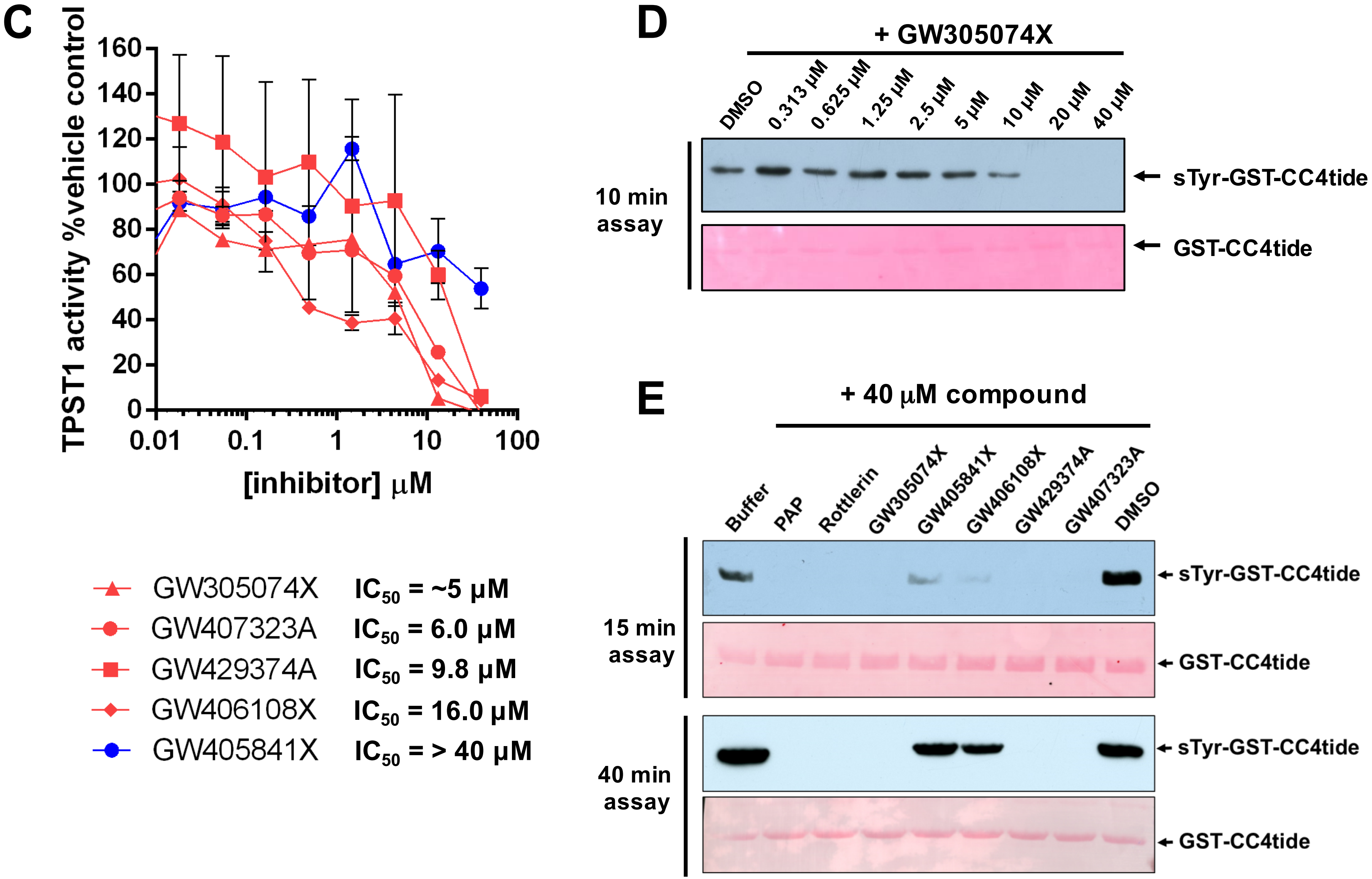

Figure 8

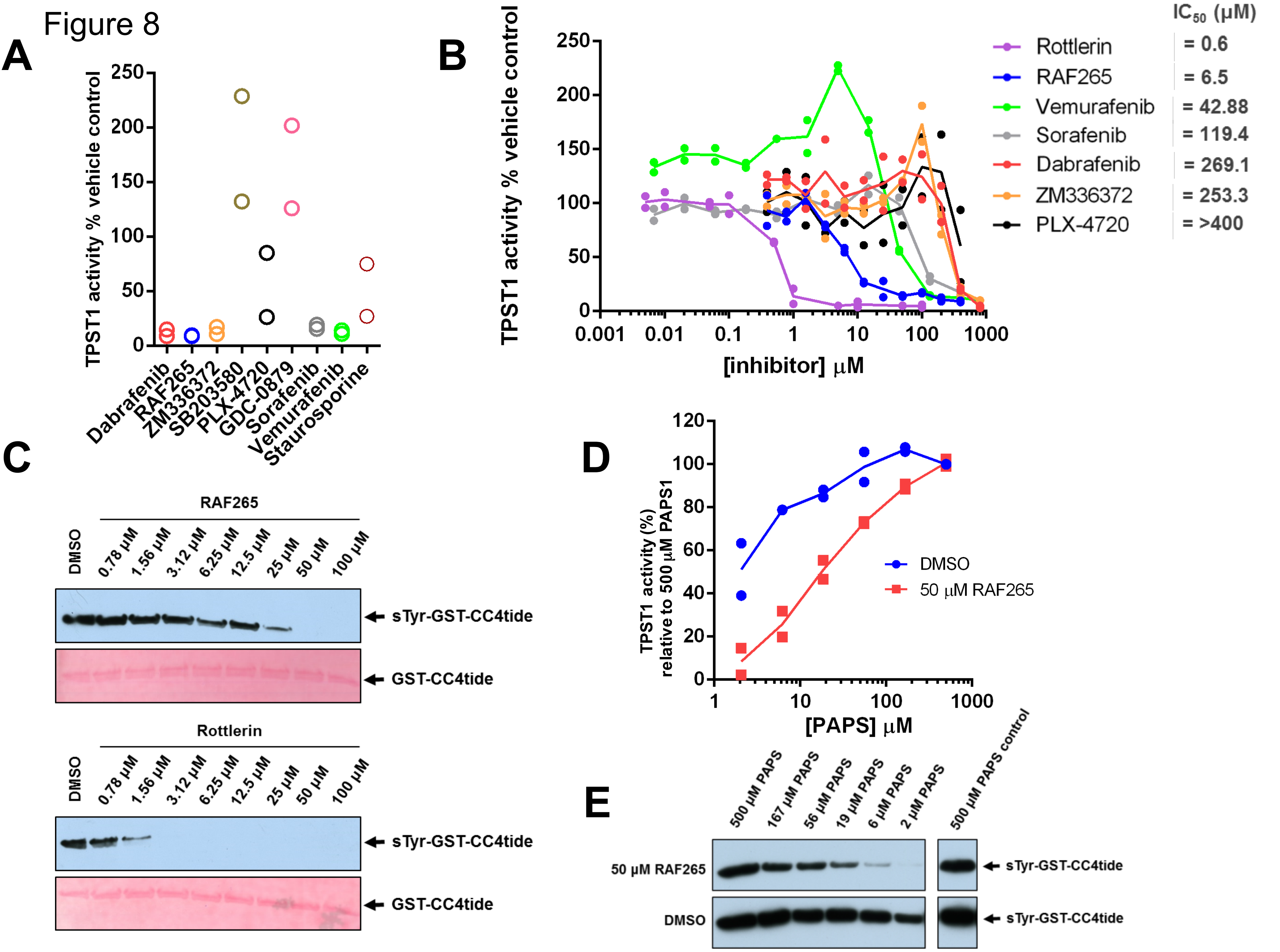
A
B
C
D
E
TPST1 activity % vehicle control
0
50
100
150
200
250
Dabrafenib
RAF265
ZM336372
SB203580
PLX-4720
GDC-0879
Sorafenib
Vemurafenib
Staurosporine
[inhibitor] µM
0.001
0.01
0.1
1
10
100
1000
IC50 (µM)
Rottlerin = 0.6
RAF265 = 6.5
Vemurafenib = 42.88
Sorafenib = 119.4
Dabrafenib = 269.1
ZM336372 = 253.3
PLX-4720 = >400
RAF265
Rottlerin
DMSO
0.78 µM
1.56 µM
3.12 µM
6.25 µM
12.5 µM
25 µM
50 µM
100 µM
sTyr-GST-CC4tide
GST-CC4tide
TPST1 activity (%) relative to 500 µM PAPS1
[PAPS] µM
DMSO
50 µM RAF265
500 µM PAPS
167 µM PAPS
56 µM PAPS
19 µM PAPS
6 µM PAPS
2 µM PAPS
500 µM PAPS control

Figure 9

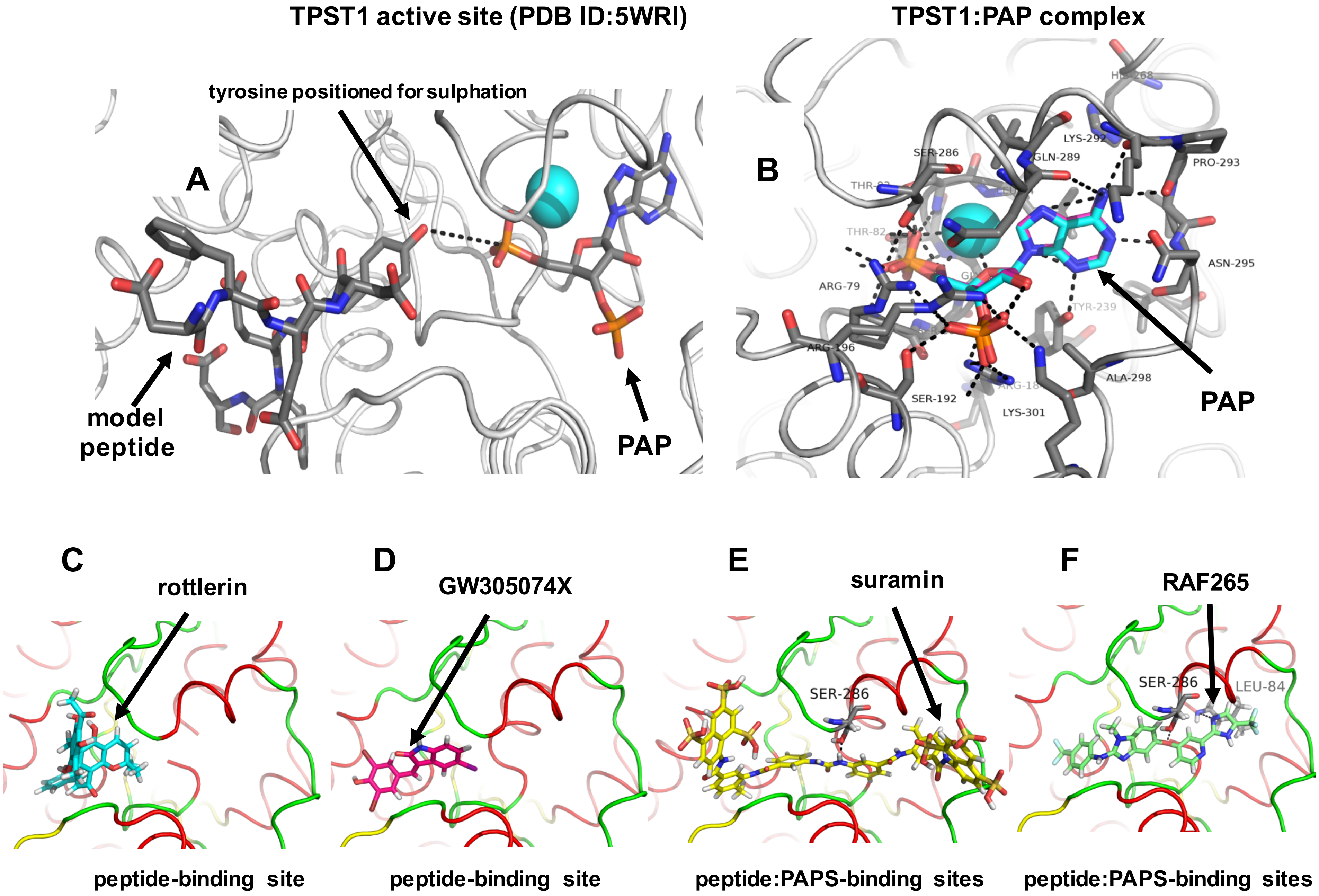
TPST1 active site (PDB ID:5WRI)
TPST1:PAP complex
A
tyrosine positioned for sulphation
model peptide
PAP
B
SER-286
THR-82
ARG-79
ARG-196
SER-192
LYS-301
LYS-292
GLN-289
PRO-293
ASN-295
TYR-239
ALA-298
PAP
C
rottlerin
peptide-binding site
D
GW305074X
peptide-binding site
E
suramin
SER-286
peptide:PAPS-binding sites
F
RAF265
SER-286
LEU-84
peptide:PAPS-binding sites

Supplementary Figure 1

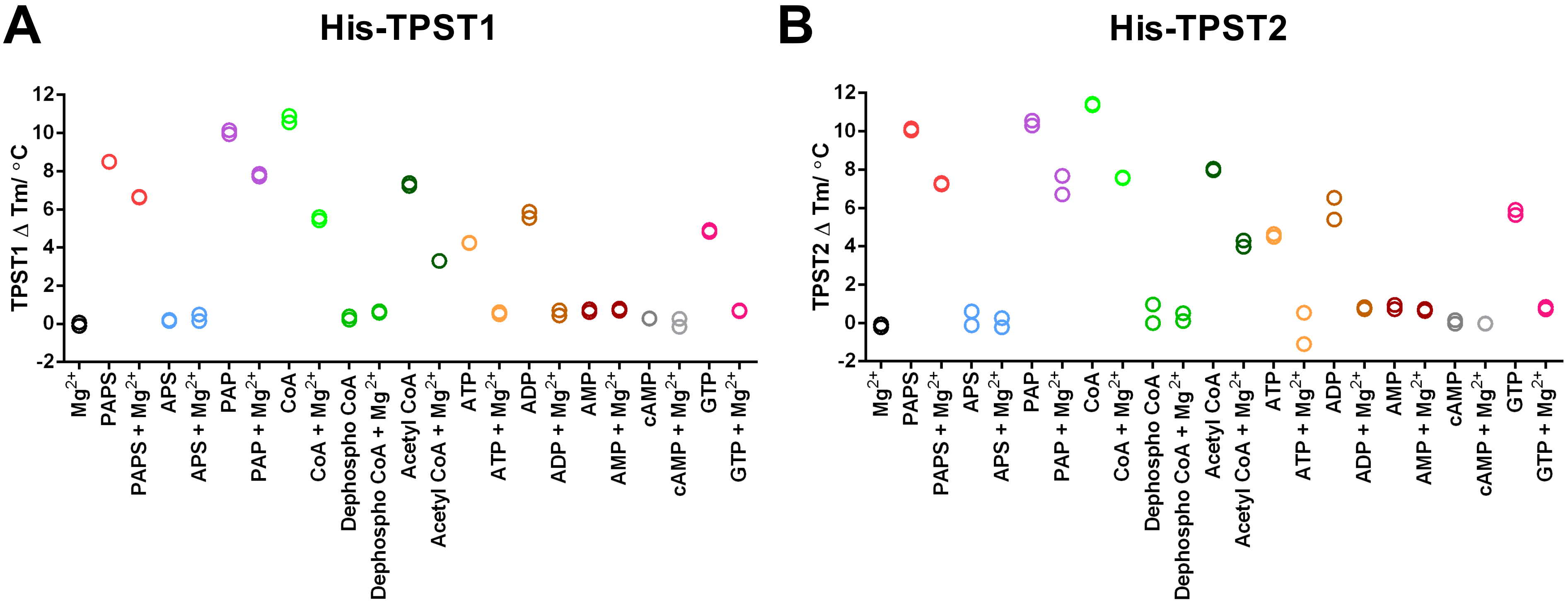

Supplementary Figure 2

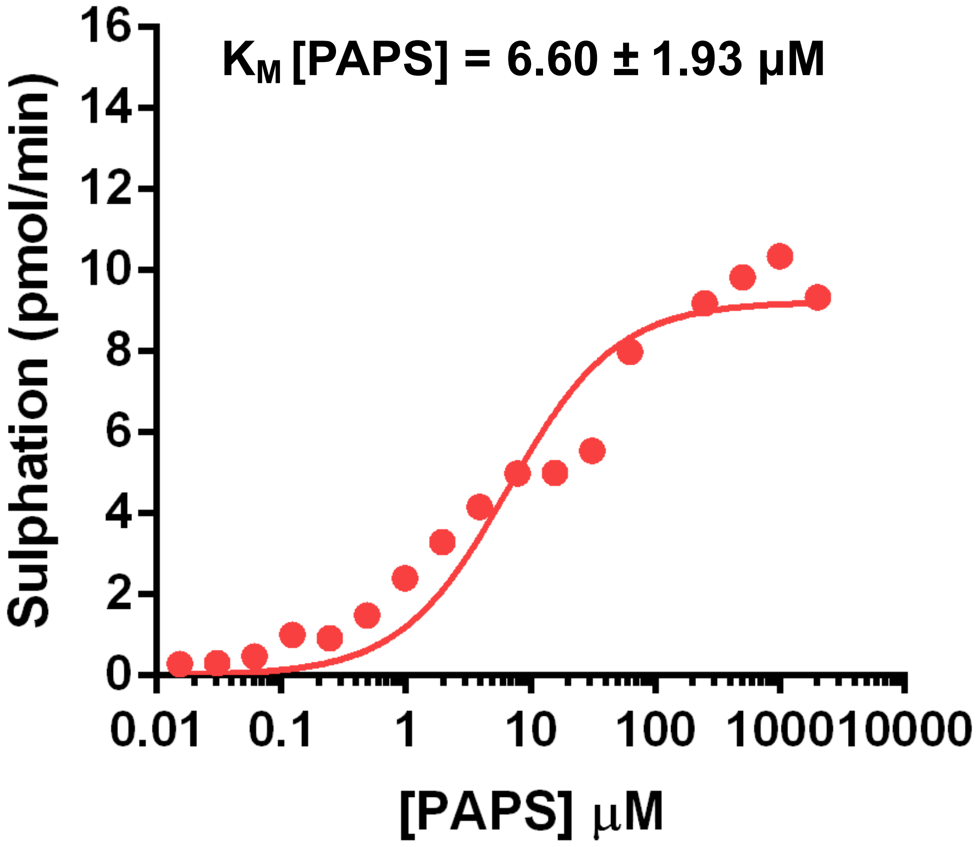

Supplementary Figure 3

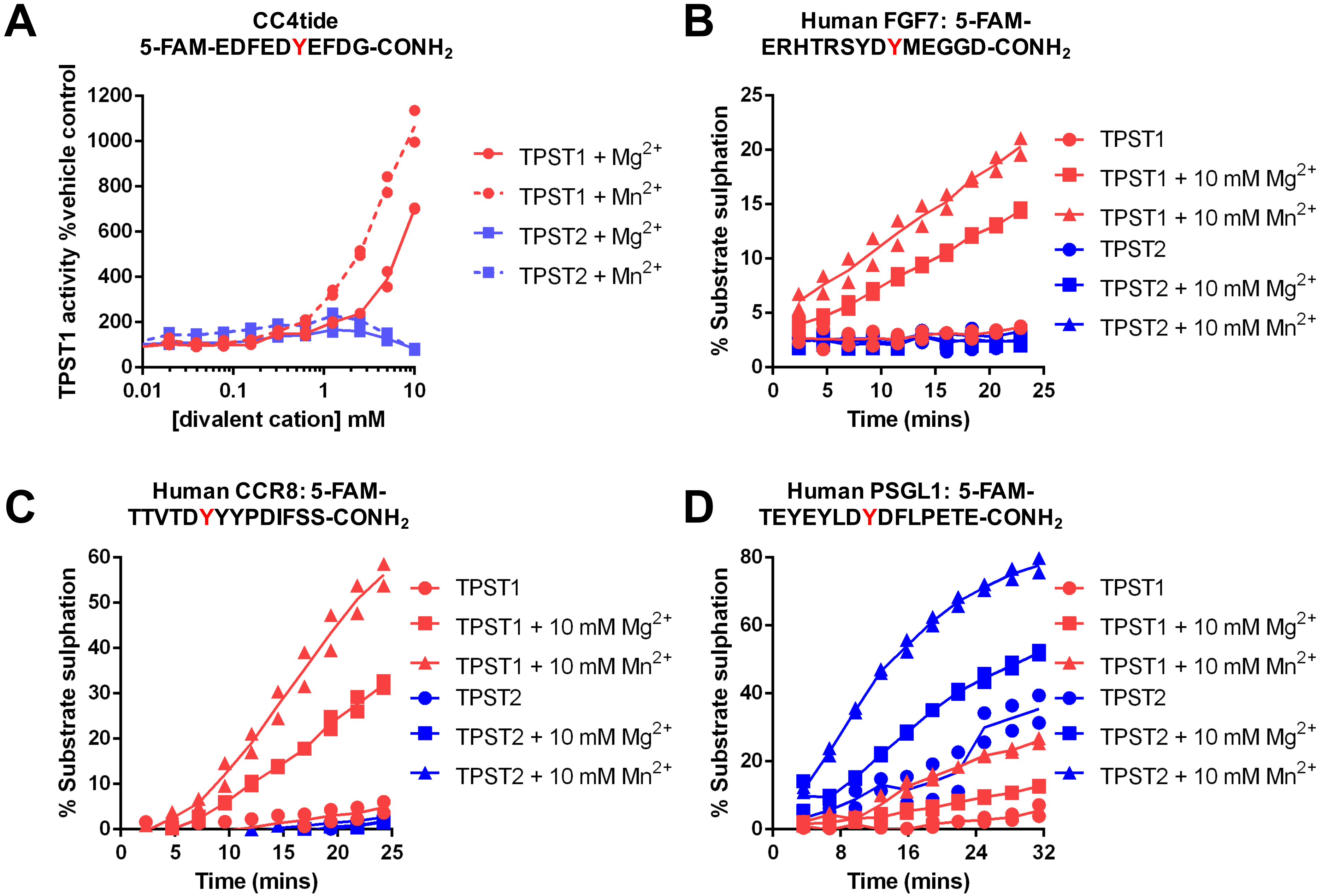

Supplementary Figure 4

rottlerin

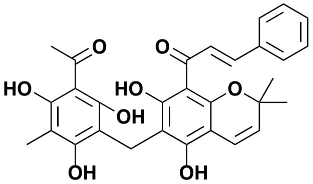

suramin

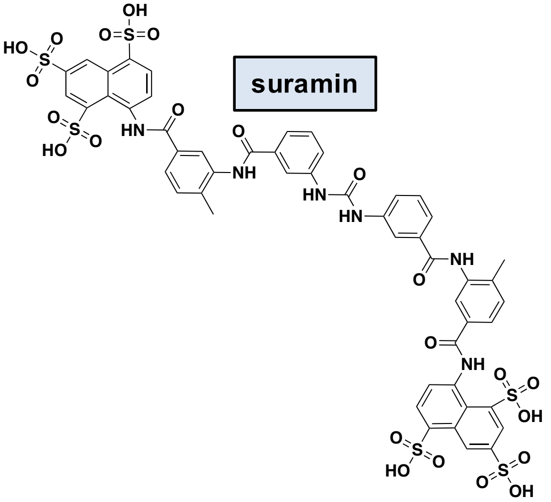

aurintricarboxylate

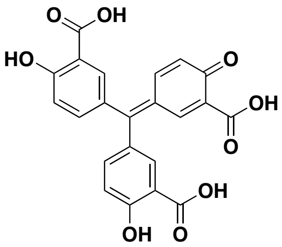

GW407323A

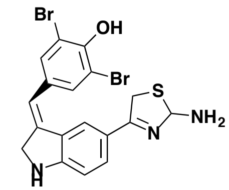

GW429374A

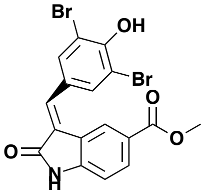

GW406108X

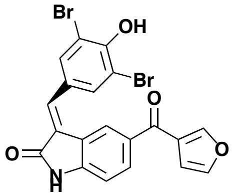

GW305074X

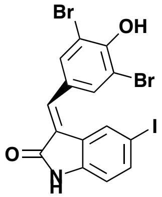

GW405841X

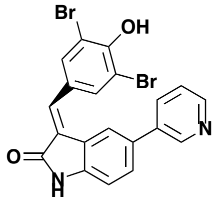

GW458344A

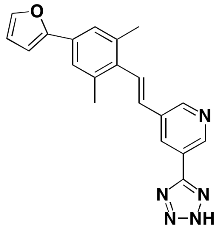

GW435821X

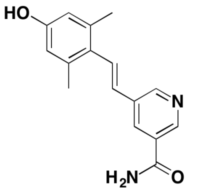

GW445017X

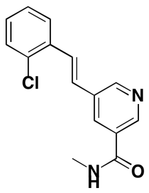

GW445015X

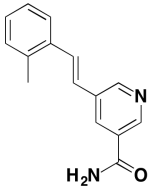

RAF265

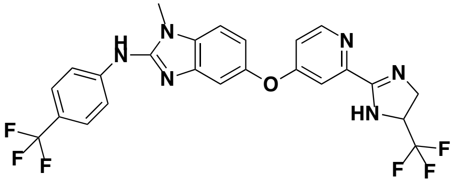

vemurafenib

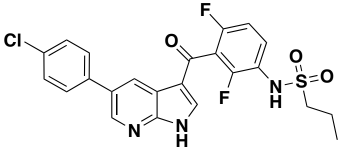

sorafenib

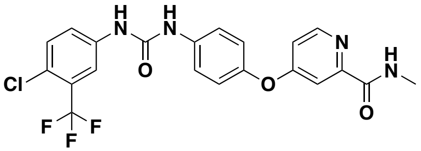

ZM336372

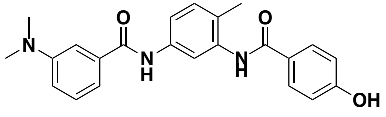

dabrafenib

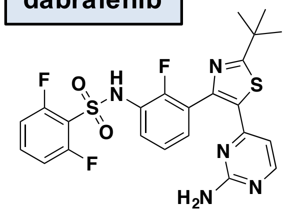

PLX-4720

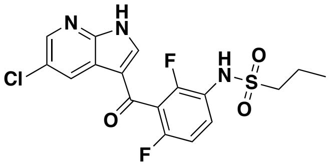

Supplement: Supplementary Figures [file BCJ-475-2435-s1.pdf]
